# *Pleurotus eryngii* as a Source of Candidate Prebiotic Substrates: Formation Routes, Potential Activities and Applications in Food Systems

**DOI:** 10.3390/foods15142527

**Published:** 2026-07-17

**Authors:** Ming Chen, Jie Chen, Yuping Zhang, Lili Zhang, Ling Xin, Ruifan Zou, Yayuan Xu, Lei Zhang

**Affiliations:** 1 Sericulture Research Institute, Anhui Academy of Agricultural Sciences, Hefei 230061, China; ming_ahas@foxmail.com (M.C.); ypzhang6330@163.com (Y.Z.); llzhang818@163.com (L.Z.); xin202509@163.com (L.X.); zouruifan0805@126.com (R.Z.); 2 Edible and Medicinal Mushroom Innovation Centre, Anhui Academy of Agricultural Sciences, Hefei 230041, China; 3 Institute of Agricultural Economics and Information, Anhui Academy of Agricultural Sciences, Hefei 230041, China; aaaskjwx@163.com; 4 Institute of Agro-Products Processing, Anhui Academy of Agricultural Sciences, Hefei 230041, China

**Keywords:** *Pleurotus eryngii*, candidate prebiotic substrate, β-glucan, gut microbiota, short-chain fatty acids, food matrix, INFOGEST, human-equivalent dose, substrate identity

## Abstract

The prebiotic concept now extends past inulin and fructo-oligosaccharides to diverse dietary compounds for selective gut microbial utilization and health promotion. This review explores *Pleurotus eryngii* as a source of prebiotic substrates, with a focus on how preparation methods and molecular traits affect its digestive stability, fermentability and food applicability. The tested materials include various mushroom-derived fractions: polysaccharides (native and modified), β-glucan extracts, powders, hydrolysates, protein co-extracts, exopolysaccharides and composite matrices. Many fractions survive upper gastrointestinal digestion and produce short-chain fatty acids whose profiles vary with the substrate during fecal fermentation. Animal studies have demonstrated their positive impacts on intestinal barriers, immunity and metabolism, and these ingredients fit well into multiple food formulations. Rather than treating *Pleurotus eryngii* as a uniform polysaccharide product, evaluations should be performed for each individual preparation. Existing data supports *Pleurotus eryngii* as a promising source of structurally diverse candidate prebiotic substrates, with a consistent biological rationale linking digestive persistence, microbial fermentation, short-chain fatty acid production, microbiota modulation and gut-related host responses. Further research is required to verify the microbiota-related causal mechanisms, assess finished food products and implement human microbiota intervention trials.

## 1. Introduction

The prebiotic concept has expanded from traditional non-digestible carbohydrates (e.g., inulin and fructo-oligosaccharides) to structurally diverse dietary substances evaluated for selective microbial utilization and host health effects [1,2]. Modern definitions stress that fermentability is insufficient for prebiotic qualification: a qualified candidate requires clear chemical composition, selective microbial utilization and verifiable health benefits. For fiber-based candidates, structural characterization, upper gastrointestinal tolerance tests, fecal fermentation analysis and human gut assessments are mandatory [3,4,5].

Beyond classical inulin-type fructans, galacto-oligosaccharides and lactulose, the pool of potential prebiotics now includes resistant starches, resistant maltodextrins, cereal and fungal β-glucans, arabinoxylans, pectin-derived oligosaccharides, mannans, polyphenol–fiber complexes and whole-food matrices [6,7,8]. Recent reviews have summarized edible and medicinal mushrooms broadly as microbiota-modulating or short-chain fatty acid (SCFA)-generating functional foods [9], but a substrate-resolved synthesis specific to *Pleurotus eryngii* that links the formation route, molecular structure, digestive fate, microbial accessibility and food-system application remains lacking.

*Pleurotus eryngii*, the king trumpet or king oyster mushroom, is a commercially important edible basidiomycete with fiber-rich composition, β-glucan variability and broad food-formulation compatibility. Its literature includes purified polysaccharide fractions and β-glucan-enriched extracts [10,11,12], together with whole-mushroom powders, chemically modified derivatives, submerged-culture exopolysaccharides, hydrolysates, protein co-extracts and composite formulations [13,14,15]. Existing human studies, by contrast, mainly address dietary feasibility, postprandial glycemia, appetite-related hormones, metabolic biomarkers and protein-digestion outcomes; integrated microbiota-mediated prebiotic validation in humans is still to come [16,17,18].

This review classifies *Pleurotus eryngii* into a group of differentiated prebiotic candidates, whose properties depend on the raw materials, preparation methods, molecular structures and food matrices. We discuss the full chain of these substrates: the formation, structural features, digestive resistance, microbial utilization, food application, design parameters and validation strategies.

Although this work follows a narrative-review framework to support a holistic, concept-oriented synthesis, a semi-structured and transparent search and selection strategy—informed by the reporting-transparency principles of PRISMA—was applied to strengthen methodological rigor. In brief, Web of Science and PubMed were searched for peer-reviewed articles published between 2000 and 2026 across six thematic blocks (composition; formation and processing; simulated digestion and fecal fermentation; gut microbiota and metabolic outcomes; food applications; and broader mushroom and prebiotic background), complemented by backward citation searching. The full search strings and eligibility criteria are provided in the Supplementary Material. As a narrative review, this study does not include protocol registration, formal risk-of-bias assessment or quantitative meta-synthesis.

## 2. *Pleurotus eryngii* as a Source of Candidate Prebiotic Substrates

### 2.1. Mushroom Biology and Composition

*Pleurotus eryngii* (DC.) Quél., an edible basidiomycete in *Pleurotaceae*, is commonly known as the king trumpet or king oyster mushroom. Commercial cultivation is extensive in East Asia and Mediterranean Europe, and the species is typically grown on lignocellulosic substrates such as sawdust–bran mixtures, cottonseed hulls, wheat straw and agricultural by-product co-substrates. Intraspecific variation is relevant to the substrate identity: reported β-glucan contents differ between *P. eryngii* var. *elaeoselini* and var. *ferulae* (22.41% versus 9.51%) [19], suggesting strains must be considered in compositional analysis.

With a thick stipe, small pileus and strong heat resistance, its fruiting body is applicable to cereals, fermented foods and meat analogs. On a dry weight basis, this mushroom is abundant in carbohydrates and dietary fibers. Its β-glucan content varies by raw material and processing: whole powders from wheat straw and wheat straw–grape marc cultivation contain 38.7% and 42.2% β-glucan [20], while aqueous extracts contain up to 48.19% β-glucan [21]. Its fiber system also includes chitin, mannans and galactans. Non-polysaccharide components (ergothioneine, phenolics, selenium-enriched protein, and UVB-induced vitamin D_2_ [22]) aid food production but are not defined as prebiotic polysaccharides without valid experimental proof.

Different from β-glucans in oats and barley (β-1,3/β-1,4 linkages), *P. eryngii* β-glucans adopt β-1,3/β-1,6 structures [23,24,25]. This feature helps them evade digestion by mammalian enzymes, making them promising microbiota-accessible prebiotic candidates.

### 2.2. Source Materials and Candidate Substrate Classes

Common raw materials for *P. eryngii* research include fruiting bodies, stipes, processing residues, submerged-cultured mycelia and in-matrix mycelia. These materials can be processed into purified polysaccharides, β-glucan extracts, mushroom powders, hydrolysates, modified derivatives, protein co-extracts, exopolysaccharides and composite products. Due to structural and processing differences, findings from one fraction cannot be extended to all mushroom-derived materials.

Stipes and industrial waste facilitate the recycling of agricultural by-products for prebiotic development. Meanwhile, mycelial growth within food matrices forms a unique route to produce functional components during food processing.

Following the current International Scientific Association for Probiotics and Prebiotics (ISAPP) framework, these criteria are adopted here as the reference standard against which each fraction is assessed: (i) a sufficiently defined chemical composition, (ii) selective utilization by host microorganisms, and (iii) a resulting, demonstrable health benefit [2]. Among these classes, purified polysaccharide and β-glucan fractions currently come closest to satisfying all three ISAPP criteria, whereas whole-mushroom powders, oligosaccharide-rich hydrolysates, protein co-extracts, exopolysaccharides and chemically modified or composite derivatives remain at an early stage.

## 3. Formation and Modulation of Microbiota-Accessible *P. eryngii* Substrates

### 3.1. Cultivation and Fermentation Routes

**Figure 1:**
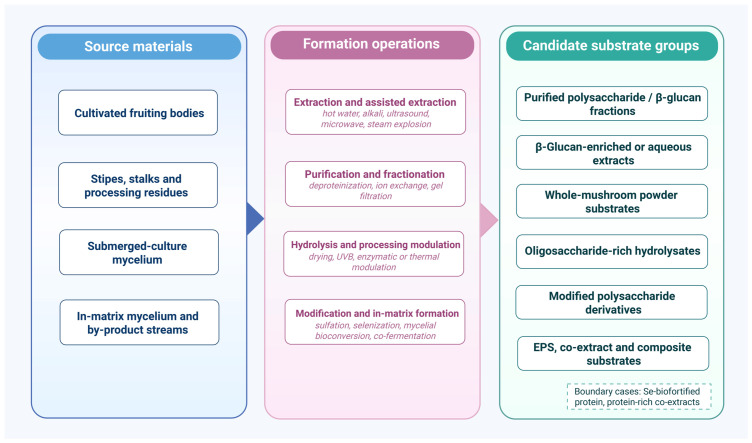
Formation routes of *Pleurotus eryngii*-derived candidate prebiotic substrates. Arrows indicate representative, non-exhaustive formation routes rather than one-to-one validated conversions between every source material, operation and substrate group.

*Pleurotus eryngii* functional components are obtained via four main pathways. Fruiting bodies are the main raw materials for polysaccharide and β-glucan extracts. Stalk residues are reused for polysaccharide production [26,27,28], and blanching broth acts as a typical processing residue [29]. Submerged fermentation generates exopolysaccharides [30], while mycelium growing inside food matrices forms new components during manufacturing [31,32]. The related routes and materials are shown in Figure 1 and Table 1.

Cultivation modes, strains and varieties alter raw material compositions [19,33,34,35]. Acid–alkali treatment recovers insoluble dietary fiber from mushroom by-products [36], and blanching broth can be developed into beverage raw materials [29].

### 3.2. Extraction and Fractionation Strategies

Hot water extraction (60–100 °C) is the mainstream method for mushroom polysaccharides, followed by precipitation and column purification [11,37,38,39,40]. Alkaline and cold water extraction are also widely used [10,26,34,41,42,43].

Various energy-assisted techniques are applied for extraction. Ultrasound produces low-molecular and strain-specific polysaccharides [19,44]. Microwave combined with supercritical CO_2_ and autohydrolysis generates oligosaccharide hydrolysates [34,45]. Steam explosion modifies substrate structures [46]. Cellulase and trypsin are adopted for auxiliary treatment [47,48]. A comparative test indicates salt extraction yields protein-rich co-extracts rather than pure polysaccharides [47].

A five-step sequential extraction obtains five structural polysaccharides from fruiting bodies, with a fresh-weight yield of 1.42% [49]. It confirms fractionation methods dominate polysaccharide structures. Standard purification steps include deproteinization and chromatography (Table 1 and Table S1). Researchers focus on whether these steps retain microbe-recognizable structures.

**Table 1:** Formation routes and key structural features of *P. eryngii*-derived candidate prebiotic substrates.

Ref.	Substrate/Fraction	Formation (Key Step)	Mw (kDa)	Key Structure Features
**A. Purified polysaccharides and β-glucans**				
[10]	APEP-A-b	Alkali + chromatography	22.5	β-1,6-glucan (β-1,3-branched)
[38]	PEP	Hot-water	NR	Glc-rich glucan
[49]	Five sequential fractions	Sequential extraction	5~2060	Five glucan/heteroglycan types
**B. β-Glucan-enriched, aqueous and crude extracts**				
[21]	M1/M2A/M2B extracts	Hot-water	NR	β-glucan-enriched extract
[50]	SPAE powder	Spray-dried	NR	Aqueous extract (α-glycosidic)
[36]	Insoluble fiber	Acid–alkali	NA	Insoluble dietary fiber
**C. Whole-powder substrates**				
[33]	PEWS/PEWSD/PEWSE	Whole powder	NR	Whole powder + β-glucan
**D. Oligosaccharide-rich hydrolysates**				
[46]	Hydrolysate extracts	Autohydrolysis	NR	Oligosaccharide hydrolysate
**E. Chemically modified polysaccharide derivatives**				
[14]	Selenized PEP	Selenized	21.6/19.7	Selenized β-glucan (triple helix)
[51]	Sulfated PEP	Sulfated	NR	Sulfated β-glucan
**F. Protein-rich co-extracts**				
[45]	Protein co-extract	Protein co-extraction	NR	Protein–carbohydrate co-extract
**G. Submerged-culture exopolysaccharides**				
[30]	EPS Fr-I/Fr-II	Submerged culture	41.0/11.1	Exopolysaccharide
**H. Boundary case: Se-biofortified protein**				
[15]	Se-protein	Alkaline (protein)	15–170 (bands)	Se-protein

Notes: Blocks are grouped by substrate class and evidence resolution, not ranked. APEP-A-b, a homogeneous branched β-1,6-glucan; PEP, *Pleurotus eryngii* polysaccharide; SPAE, *Pleurotus eryngii* aqueous extract; PEWS, *Pleurotus eryngii* in whole food matrix form; PEWSD, *Pleurotus eryngii* in in vitro digested form; PEWSE, *Pleurotus eryngii* form rich in β-glucans extract; EPS, exopolysaccharide. NR, not reported. NA, not applicable. Yields are dry-weight percentages unless otherwise specified; submerged-culture EPS yields are reported in g/L; Mw values are reported in kDa unless otherwise specified and are retained as reported. The complete structural inventory (formation routes, yields, Mw, monosaccharide ratios, linkage, branching, and degree of substitution) is provided in Supplementary Table S1.

### 3.3. Processing-Induced Structural Modulation

Drying strongly affects the structure of *Pleurotus eryngii* products during preparation and storage. A spray-dried aqueous extract (SPAE; 180 °C inlet, 90 °C outlet) has shorter polymer chains and more α-glycosidic bonds (843 cm^−1^) than its freeze-dried counterpart [50]. In soymilk, oven-dried *P. eryngii* polysaccharide (ODPEPS) forms irreversible Maillard aggregates, whereas the freeze-dried and boiling-treated *P. eryngii* polysaccharides (FDPEPS and BTPEPS) stay soluble [47]. Drying acts as a structural regulation step instead of routine preservation.

Physical and thermal pre-treatments also modify substrate structures. Steam explosion creates porous steam explosion *Pleurotus eryngii* polysaccharides (SEPEP) [48]. Autohydrolysis yields oligosaccharide-rich hydrolysates [46]. Ultrasound breaks polysaccharides into low-molecular fragments (3.2 kDa and 2.0 kDa) [44], and microwaves cause strain-dependent yield changes with unclarified structural effects [34].

Chemical modification optimizes material performance. Sulfation (DS: 0.12–0.92) improves polysaccharide water solubility [51,52]. Selenization slightly reduces molecular weight while maintaining triple-helix structures and high selenium content [15]. Cultivation-based selenium enrichment produces Se-containing proteins [14]. These two types of selenium-enriched products must be differentiated.

Drying, pre-treatment and chemical modification all actively alter material properties for digestion and fermentation analyses.

### 3.4. Food Processing as a Formation Route

Unlike conventional substrate preparation, food processing can alter the composition, structure, and solubility of *Pleurotus eryngii*-derived ingredients. While a range of processing techniques have the potential to affect gastrointestinal stability, corresponding validation using finished food products remains limited.

Mycelial bioconversion represents a typical form of in situ substrate transformation. In 3D-printed potato starch matrices, mycelial growth reduced starch content from 84.18% to 60.35% while increasing β-glucan content from 12.57% to 24.31% [31]. In 4D-printed meat analogs formulated with 6% mycelia, 10 days of incubation at 25 °C and 95% relative humidity (RH) increased the dry weight of the mycelia to 18.2% of the total and enhanced sample hardness 6.11-fold [32]. Such metabolic activity of mycelia drives substantial modification of mushroom-derived components within food matrices.

Food processing can also drive the formation of de novo bioactive compounds. For instance, UVB irradiation applied during baking elevates vitamin D_2_ content in mushroom chips nearly 13-fold, from 0.593 to 7.50 μg/g [22]. Notably, this conversion relies on endogenous sterols in mushrooms, rather than on prebiotic polysaccharide substrates.

Matrix-induced molecular aggregation is another common processing-induced alteration. Oven-dried PEPS forms insoluble Maillard aggregates when incorporated into soymilk, whereas freeze-dried and boiled PEPS samples retain their solubility [47]. Microbial fermentation likewise modifies mushroom extracts and polypeptides, but the associated structural modifications have yet to be systematically characterized [53,54].

The aforementioned changes stem from three distinct mechanisms: mycelial bioconversion remodels the structure of prebiotic substrates; UV irradiation generates de novo bioactive compounds; and molecular aggregation modulates substrate accessibility without covalent bond cleavage. Distinguishing among these mechanisms is critical for interpreting findings from food application studies. Most existing studies on mushroom-enriched foods evaluate only processing performance and gross product composition, neglecting post-processing structural changes to the functional mushroom-derived components.

These processing-driven modifications directly shape the subsequent digestive fate and microbial utilization of mushroom components. Thermal aggregation, Maillard conjugation, protein–polysaccharide complexation, and low-water-activity baking can all immobilize mushroom-derived carbohydrates, reducing their accessibility during simulated digestion and fecal fermentation. UVB treatment primarily produces vitamin D_2_ from endogenous mushroom sterols; it should not be interpreted as generating prebiotic substrates unless independent evidence confirms preserved or enhanced carbohydrate accessibility. Similarly, microbial fermentation and in-matrix mycelial growth can remodel carbohydrate structure, but the colonic accessibility of the final processed matrix cannot be predicted from the properties of the starting raw material. Accordingly, processed food matrices must undergo full re-characterization before any interpretation of gut-targeted digestion and fermentation results.

## 4. Structural Features, Physicochemical Properties and Digestive Fate

### 4.1. Molecular Weight, Monosaccharide Composition and Linkage Patterns

*Pleurotus eryngii* polysaccharides vary greatly in molecular weight (Mw). Their properties rely on molecular size, monosaccharide composition and glycosidic linkages. Ultrasonic extracts are low-molecular fractions [44], and APEP-A-b is a typical medium-sized β-1,6-glucan [10]. Large *P. eryngii* polysaccharide (PEP) fractions and stipe-derived glucans extend to megadalton scales [12,26,37]. Most PEP fractions are dominated by glucose-rich β-glucans [10,12,37,38]. Galactans, mannans, heteroglycans and mixed α/β-glucans are also common components [11,14,35,42,43,49]. Molecular weight alone cannot determine microbial accessibility or biological functions.

Glycosidic linkages effectively differentiate similar polysaccharides. β-1,3 and β-1,6 linkages prevail across most fractions [27,39,41,51,55], and α-linked polysaccharides constitute key subgroups [26,35,49]. Key structural parameters are listed in Table 1, and the complete set is provided in Supplementary Table S1.

### 4.2. β-Glucan Architecture and Heteroglycan Diversity

Three main structural groups of characterized β-glucans exist: mushroom-type β-1,3-glucans with single β-1,6 branches [55]; β-1,6-backbone polysaccharides bearing β-1,3 substitutions (APEP-A-b as a representative) [10]; and high-Mw β-1,3/1,3/1,6 glucan–protein conjugates with sparse α-1,6-Gal branches [26,41]. Sulfation preserves native skeletons with glucose-based substitution degrees of 0.12–0.92 [51], whereas galactose-containing analogs show C6-specific sulfation (DS = 0.69) [52].

Only hot-water extracts, aqueous extracts and stepwise fractions have undergone direct β-glucan quantification [21,33,49]. Most research identifies β-glucans indirectly via monosaccharide and linkage profiling. Fungal β-1,3/1,6-glucans are structurally distinct from cereal β-1,3/1,4-glucans and must not be conflated.

*P. eryngii* accumulates various heteroglycans, including methylated α-1,6-galactan WPEP-N-b [40], low-Mw α-D-galactans for mastitis research [11], and β-1,4-Glcp/β-1,3,6-Manp mannoglucan KOMAP [43]. Single substrates yield multiple structurally divergent polysaccharide fractions [35,49], so Table 1 reports individual fractions rather than broad species summaries. Triple-helix and rigid-rod conformations are documented for these polymers [14,30,42].

Beyond serving as chemical descriptors, these structural attributes are expected to govern microbial accessibility, fermentation kinetics and microbiota selectivity [8,56]. Lower-Mw hydrolysates or ultrasound-fragmented fractions may show improved solubility and faster microbial degradation through the increased exposure of catalytic sites, but depolymerization could also alter microbial selectivity by eliminating taxon-specific higher-order structural motifs [57]. Because the fungal β-1,3/β-1,6 architecture differs fundamentally from the β-1,3/β-1,4 backbone of cereal β-glucans, the two likely engage different gut glycoside-hydrolase repertoires and, in turn, different microbial utilizers [56]. Branch density is a further factor, shaping conformation, solubility and the availability of enzyme-binding sites [57]. Higher-order assemblies, triple-helix structures, rigid-rod conformations and spontaneous or Maillard-derived aggregates may restrict enzyme access, delay fermentation onset or shift fermentation toward the distal colon, whereas random-coil conformations generally enhance degradability [57]. Chemical modifications such as sulfation and selenization may additionally alter utilization through changes in molecular charge and solubility, although supporting data for modified *P. eryngii* glucans remain sparse.

### 4.3. Solubility, Particle Morphology and Physicochemical Behavior

Solubility and morphology vary with extraction and processing. Cold-water, hot-water and alkaline fractions differ in solubility range and aggregation behavior [42,49], while gel-like β-1,3/1,6-glucan-rich material illustrates the poorly soluble end of the spectrum [55]. Drying can further change behavior: spray-dried aqueous extract showed spherical particles and shorter polymer chains than freeze-dried PEP [50], and oven-dried PEPS formed insoluble Maillard aggregates in soymilk whereas freeze-dried and boiling-treated preparations remained dispersible [47].

Several preparations illustrate how morphology and bulk-fiber properties differ from soluble-polysaccharide behavior. Steam-explosion-assisted extraction produced porous SEPEP material [48], submerged-culture EPS fractions differed in conformation and flexibility [30], and acid–alkali-derived insoluble fiber displayed water-holding, swelling and crystalline-cellulose features [36]. Autohydrolysis generated an oligosaccharide-rich hydrolysate and is best interpreted as a distinct substrate class [46]. Chemical modification, including sulfation and selenization, changed solubility, selenium content or conformation while maintaining the need to distinguish modified derivatives from parent polysaccharides [14,51,52].

### 4.4. Resistance to Simulated Upper Gastrointestinal Digestion

Direct evidence verifying the resistance of *Pleurotus eryngii* polysaccharides to upper gastrointestinal digestion has been provided in an existing study [38]. Hot-water-extracted PEP exhibited no measurable changes in molecular weight and no release of free monosaccharides after exposure to simulated salivary, gastric, and small intestinal fluids. This fraction was subsequently fermented by human fecal microbiota, with a concomitant drop in pH and accumulation of SCFAs.

The widely used INFOGEST digestion protocol was developed from the original international consensus static digestion method [58] and its updated 2.0 version [5]. Building on this standardized framework, studies employing the INFOGEST 2.0 system have further explored the associations between digestive metabolites, fermentation products, and intestinal mucus barrier biomarkers [59,60]. Collectively, these findings indicate that specific *Pleurotus eryngii* polysaccharides can survive upper gastrointestinal transit and serve as fermentable substrates for colonic microbes, which supports the prebiotic candidacy of these fungal polysaccharides. However, the presence of protein-rich co-extracts, hydrolysates, selenium-enriched proteins, and oat β-glucan composites introduces ambiguity in attributing the observed effects to polysaccharides alone [15,46,61,62]. Furthermore, food formulation and processing may alter the solubility and aggregation state of polysaccharides in final products. For these reasons, simulated digestion is regarded as a necessary preliminary assessment of prebiotic potential, rather than definitive evidence of prebiotic activity.

Given the inconsistent application of the INFOGEST 2.0 protocol, cross-study comparisons of digestive resistance remain largely qualitative. When comparing results across digestion studies, key methodological variables must be accounted for: inclusion of the oral phase, enzyme source and activity, pH control conditions, bile concentration, digestion duration, static versus dynamic design, and whether digesta are subsequently subjected to fecal fermentation. The digestive fate of polysaccharides should be evaluated for each substrate and food matrix individually, rather than inferred solely from the source species.

### 4.5. From Digestive Resistance to Microbial Accessibility

Selected *P. eryngii* polysaccharides appear able to resist upper-gastrointestinal digestion long enough to remain available for microbial fermentation. This availability is necessary but not sufficient: whether a resistant polysaccharide is actually fermented, and by which taxa, depends jointly on its structural features and the carbohydrate-degrading enzyme repertoires of the resident gut microbiota [56]. This inference is strongest for materials tested directly in digestion–fermentation sequences. It should be re-evaluated when extracts are hydrolyzed, co-extracted with protein, chemically modified or incorporated into foods.

## 5. Potential Prebiotic Activities: Fermentation, Microbiota Modulation and Gut-Linked Effects

### 5.1. Fecal Fermentation and Metabolite Outputs

**Figure 2:**
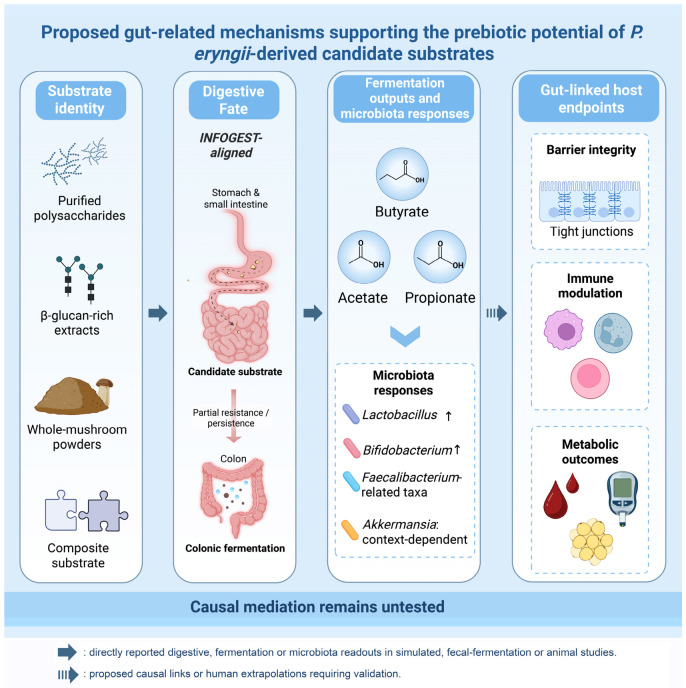
Proposed gut-related mechanisms supporting the prebiotic potential of *Pleurotus eryngii*-derived candidate substrates.

Figure 2 outlines a sequential gut-relevant evidence chain: polysaccharides first survive upper gastrointestinal digestion and become available to colonic microbes, followed by fermentative metabolite production, intestinal microbiota remodeling and downstream host physiological outcomes. Table 2 summarizes the distinct categories of supporting evidence, while Supplementary Table S2 lists all the included research records in full.

Monoculture screening assays preliminarily assess the microbial degradability of mushroom polysaccharides. Glucans isolated from mushroom stems stimulate the proliferation of *Lactobacillus* and *Bifidobacterium* strains [26]; dried PEPS fractions boost the growth of *Bifidobacterium longum* and trigger medium acidification [47]; gamma irradiation alters the growth curve of crude polysaccharides when cultured with *L. plantarum* [63]. Such single-strain tests rapidly screen polysaccharide candidates for subsequent mixed fecal fermentation assays.

Fecal fermentation systems further characterize substrate utilization by complex intestinal microbial consortia. Both PEP and carbohydrate–protein co-extracts can be fully fermented by human fecal microbiota [38,45]. Aqueous extracts and β-glucan-enriched fractions produce fermentation profiles highly dependent on the individual donor microbiota composition [20,21,33]. Whole-mushroom powders and composite blended samples expand the scope of tested substrates [59,60,62]. Extended experimental designs combining simulated digestion, fecal fermentation and intestinal mucus co-culture, cross-mushroom comparative trials, and fecal samples from specific population groups broaden the range of detectable biological endpoints [64,65,66,67,68]. Short-chain fatty acids (SCFAs) serve as key signaling molecules mediating gut-host crosstalk. Their biological effects cannot be judged solely by total yield; the molar ratio of acetate, propionate and butyrate, intestinal absorption efficiency and target tissue microenvironment collectively determine their physiological functions [69,70].

### 5.2. SCFA Production, pH Reduction and Fermentation Metabolites

*P. eryngii* materials function as fermentable fibers during in vitro fermentation. Preparations including *P. eryngii* polysaccharide, aqueous extracts, carbohydrate–protein extracts, and the purified β-1,6-glucan fraction APEP-A-b all drive SCFA production and reduce pH [10,21,38,45]. This pattern aligns with in vivo findings: diets supplemented with various mushroom preparations uniformly elevate intestinal SCFA concentrations in animal models [12,28,50,71,72,73]. While the dominant SCFA species varies across sample types, dosages, and animal models, enhanced SCFA synthesis represents a consistent effect across these structurally distinct fractions.

Extensive prior research has established SCFAs as key mediators of epithelial homeostasis, immune modulation, and metabolic regulation. Their effects are exerted through two primary mechanisms: signaling via G-protein-coupled receptors (FFAR2/FFAR3 and GPR109A) and inhibition of histone deacetylases, both supported by causal evidence from receptor knockout and metabolite supplementation studies [69,74,75]. Against this established mechanistic framework, it is biologically plausible that SCFA production from *P. eryngii* substrates contributes to the observed host benefits, even though this specific causal pathway has not been directly validated for *P. eryngii*-derived materials.

Acetate, propionate, and butyrate each play distinct roles in epithelial, immune, and metabolic regulation; however, most studies of *P. eryngii* only document correlated changes in SCFA levels, microbiota composition, and host endpoints, rather than employing study designs that formally test mediating effects. Establishing causality would require targeted approaches, including matched non-fermentable controls, interrogation of SCFA receptors or downstream pathways, microbiota transfer experiments, isotope tracing, and human trials linking defined substrate intake to alterations in microbiota, metabolites, and clinical endpoints.

Metabolomic analyses reveal additional bioactivities independent of SCFA production. Low-molecular-weight α-D-galactan heteroglycan, selenium-enriched proteins, and protein–oat β-glucan composites modulate kynurenine, vitamin, amino acid, and organic acid metabolism, and also mediate microbial lead adsorption [11,15,62]. Each component exhibits distinct metabolic profiles, meaning their biological functions cannot be attributed to a single shared mechanism.

### 5.3. Microbiota Modulation in Animal Models

In vivo evidence for the prebiotic candidacy of *P. eryngii* preparations derives from their ability to remodel gut microbiota toward beneficial taxa across animal disease models. The published research covers obesity/diabetes induced by high-fat diets with extracts, mushroom powders or composite foods [50,71,72,73,76], toxicant-challenged animals receiving protein–polysaccharide mixtures [15,28,77,78,79], and DSS colitis plus mastitis models [11,13]. No single fixed microbial profile emerges; instead, the polysaccharide category, dosage and disease state collectively determine the enriched beneficial taxa.

*Akkermansia muciniphila* serves as a typical example, with its abundance modulated by the experimental context rather than showing a universal upward trend, consistent with prior microbiome reports [80,81]. Aqueous extracts and whole-mushroom powders raise its abundance [50,71,72], alongside crude polysaccharides and extruded rice composites [73,79]. In high-fat diet models, this enrichment aligns with the bacterium’s established roles in mucin-layer maintenance and intestinal barrier integrity [80], offering a plausible mechanistic link to the observed improvements in metabolic homeostasis [82]. The downregulation of *Akkermansia* observed under DSS colitis is not conflicting but biologically reasonable: WPEP simultaneously upregulated *Bifidobacterium pseudolongum*, *Lactobacillus reuteri*, *Ligilactobacillus salivarius* and *Ruminococcus bromii* [13], a taxonomic rearrangement driven by mucosal injury, substrate structure and baseline gut microbes. *Lactobacillus*-related populations display the most stable enrichment signals across β-glucan/powder trials [10,12,71] and colitis/toxicity models [13,73,76,79], in keeping with their broad anti-inflammatory and epithelial barrier-protective functions, whereas *Bifidobacterium* enrichment is detectable yet less consistent [10,72], likely explained by inter-study differences in baseline microbiota composition and disease severity. Butyrate-producing taxa such as *Roseburia* show similarly context-dependent responses, with their implications for colonic health dependent on the local microbial ecosystem and experimental conditions.

These mushroom fractions also restrain opportunistic pathogens: *Escherichia-Shigella*, *Desulfovibrionaceae*, *Cryptobacteroides*, *Streptococcus* and *Clostridium perfringens* all declined in colitis, toxic and immune-related animal assays [10,73,76,77]. Shifts in Firmicutes/Bacteroidota ratios have been recorded in several studies [11,28,71,79], though this ratio only offers descriptive information instead of acting as a gut health biomarker [83]. Individual microbial shifts depend on substrates and experimental backgrounds, but the consistent dual function of specific *P. eryngii* bioactives—promoting beneficial commensals and suppressing opportunists—matches a core expectation for candidate prebiotics.

### 5.4. Barrier-, Immune- and Inflammation-Related Effects

Microbial community shifts induced by *P. eryngii* preparations elicit measurable host intestinal responses. Tight junction and mucin biomarkers provide evidence consistent with gut barrier maintenance: protein extracts, crude polysaccharides, stalk polysaccharides and selenium-rich proteins restored key barrier proteins in colitis, high-fat, aflatoxin and lead toxicity models [15,38,73,79]. Ex vivo fermentation supernatant and intestinal biopsy assays replicated such barrier and mucus protective effects [84,85,86], while combined digestion–fermentation assays for PEP established a direct link between polysaccharide fermentation and mucus secretion [49,53].

Immune markers align with barrier-protective phenotypes yet exhibit stronger model dependence. Reduced IL-1β, IL-6, TNF-α and inflammasome signals, alongside higher IL-10, are commonly detected across diverse experimental setups [13,38,71,73,79]. PEP, APEP-A-b and low-Mw heteroglycans further exert regulatory effects on immune organs, macrophages and mastitis lesions [10,11,12]. Notably, one fermentation supernatant elevated TNF-α [87], indicating cell culture and challenge conditions may reverse inflammatory readouts, rather than negating the general anti-inflammatory characteristic of these mushroom-derived substrates.

### 5.5. Systemic Metabolic Phenotypes and Outstanding Validation Requirements for Prebiotic Capacity

Consistent with the gut barrier, immune and inflammatory responses outlined above, *Pleurotus eryngii* bioactive fractions trigger gut microbial shifts that correlate with favorable systemic metabolic phenotypes in animal models. Most metabolic research adopts high-fat diet (HFD) and HFD/STZ diabetic rodent systems. PEPF, PEP, insoluble mushroom fiber, whole fruiting-body powder, crude polysaccharide extracts and extruded rice composite formulations all improved lipid homeostasis, body weight control and glycemic regulation, alongside clear remodeling of key intestinal bacterial taxa [28,71,72,73,76,88]. Beyond metabolic syndrome models, complementary disease systems broaden the spectrum of gut-linked physiological readouts: low-molecular-weight heteroglycans alleviate mastitis symptoms [11], while selenium-enriched proteins and selenium-biofortified mushroom powders mitigate lead toxicity through intestinal lead adsorption [15,65]. Such observations connect the in vitro fermentative traits of mushroom polysaccharides to tangible whole-body physiological benefits, forming a complete line of evidence spanning fermentation, microbiota reshaping and downstream gut-dependent host effects.

Integrating all layers of the data covering polysaccharide fermentation, intestinal microbiota remodeling, epithelial barrier maintenance, immune homeostasis and metabolic regulation yields a unified conclusion: structurally defined, purified *P. eryngii* bioactive fractions redirect gut microbiota toward health-beneficial configurations. This microbial regulatory capacity supports the prebiotic candidacy of compounds isolated from *P. eryngii*, which aligns with the review’s core theme linking fermentability, microbiota modulation and gut-mediated systemic effects. Two critical unresolved research gaps still distinguish preliminary prebiotic potential from definitive clinical validation. First, it remains unconfirmed whether intestinal microbes or their fermentative metabolites act as the causal mediator of the observed host protective phenotypes. Second, it is unknown whether fully characterized, standardized mushroom substrates can replicate these beneficial gut and systemic effects in human trials centered on gut microbiota readouts. Although existing investigations of mushroom-fortified meals, functional snacks and purified β-glucan supplements verify that *P. eryngii* raw materials can be incorporated into diets at biologically relevant dosages [16,17,18], these published works fail to resolve either of the two fundamental validation questions.

**Table 2:** Digestive fate, fermentation and microbiota-related activities of *P. eryngii*-derived substrates.

Ref.	Substrate/Preparation	Model/Exposure	Main Microbiota, Metabolite and Host Readouts	Relevance to Candidate-Prebiotic Framing
**A. In vitro fecal fermentation studies**				
[38]	PEP (Glc/Man/Gal 78.32/9.43/8.47%; Mw NR; β-glucan NR)	Simulated saliva/gastric/intestinal digestion; 24 h human fecal fermentation, 1.0 g PEP; donor context: 5 healthy human fecal donors	Microbiota/metabolites: No Mw/free-monosaccharide change during upper digestion; pH 7.64 → 5.62; total SCFA 52.86 ± 2.01 mM; enriched Firmicutes incl. *Enterococcus*/*Streptococcus*/*Clostridium*; reduced Proteobacteria/Bacteroidetes	Microbiota-accessible substrate (human fecal-fermentation evidence)
[33]	PEWS/PEWSD/PEWSE (β-glucans 38.7/39.8/49.7%)	Simulated digestion; 24 h elderly human fecal fermentation; donor context: 5 healthy older-adult fecal donors	Microbiota/metabolites: All forms increased total SCFA, acetate, propionate and butyrate; whole matrix enriched *Bifidobacterium* spp. and *Faecalibacterium prausnitzii*; *Bacteroides* spp. increased across forms	Candidate prebiotic (elderly fecal-fermentation evidence)
[62]	*P. eryngii* protein–oat β-glucan complex; protein purity 60.07%	INFOGEST digestion; 48 h fecal fermentation; 2:1 protein–oat β-glucan complexes; donor context: 7 healthy volunteers	Microbiota/metabolites: W-PEP-OG showed strongest digestion resistance and best overall fermentation profile; host/barrier/immune: no in vivo host endpoint	Composite-system evidence (protein–oat β-glucan complex; attribution to mushroom β-glucan is limited)
[64]	Freeze-dried whole powder (58.3% carbohydrates; 20.7% protein)	Sequential in vitro digestion + 48 h fecal fermentation; donor context: human fecal donor n NR	Microbiota/metabolites: Lowered pH; generated lower total SCFA than blank; promoted Actinobacteria/*Bifidobacterium* and Bacteroidetes; reduced Proteobacteria/Fusobacteria/Firmicutes	Microbiota-modulating whole-food substrate (whole-powder fecal-fermentation evidence; SCFA profile context-dependent)
**B. In vivo animal studies**				
[10]	APEP-A-b (22.5 kDa branched β-1,6-glucan; 90.1% Glc)	Simulated digestion; 24 h mouse fecal fermentation; mice 100/200/500 mg/kg·d for 14 d; donor context: mouse fecal donor n NR	Microbiota/metabolites: No obvious Mw change in digestion; fermented/degraded by fecal microbiota; 200 mg/kg increased *Lactobacillus*, *Bifidobacterium*, Lachnospiraceae, Rikenellaceae; decreased *C. perfringens*; increased cecal acetate and butyrate; host/barrier/immune: 200 mg/kg increased phagocytosis capacity by 14.8%; 100/500 mg/kg no obvious change	Candidate prebiotic (mouse digestion–fermentation and in vivo evidence; dose response noted)
[13]	WPEP (167 kDa β-type glycosidic polysaccharide; Xyl/Man/Glc/Gal 21.35/3.28/73.22/1.63)	DSS colitis mice; 0.2/0.8 g/kg gavage for 44 d; donor context: NA	Microbiota/metabolites: 0.8 g/kg decreased *Akkermansia muciniphila* and *Clostridium cocleatum*; increased *Bifidobacterium pseudolongum*, *Lactobacillus reuteri*, *Ligilactobacillus salivarius*, *Ruminococcus bromii*; host/barrier/immune: DAI 2.78 → 1.80; colon length 9.31 → 10.89 cm; reduced mucosal immune cells and cytokines	Candidate prebiotic (mouse colitis model with microbiota, barrier and immune readouts)
[15]	Se-enriched protein (360.64 mg/kg Se; bands 15–170 kDa)	Simulated digestion; Caco-2; fecal fermentation; mice 200 mg/kg/d in lead toxicity; donor context: fecal donor n NR	Microbiota/metabolites: Digest hydrolysis degree 27.65%; fermentation-enriched *Megasphaera*/*Mitsuokella*/*Phascolarctobacterium* and decreased *Escherichia*/*Fusobacterium*; microbial Pb adsorption 90.12%; host/barrier/immune: restored colonic tight junction genes; lowered IL-6/TNF-α; reduced tissue lead accumulation	Non-polysaccharide boundary case (Se-enriched protein in digestion, fermentation and lead-toxicity model)
[72]	Whole powder (35.7% dietary fiber; 1/3% *w*/*w* HFD diet)	HFD mice for 8 weeks; donor context: NA	Microbiota/metabolites: 3% powder increased cecal acetate 41.77%, butyrate 130.44%, isobutyrate 52.38%, valerate 109.57%, isovalerate 71.57%; enriched *Akkermansia*/*Lactobacillus*/*Bifidobacterium*/*Sutterella*; host/barrier/immune: reduced weight/fat gain, serum lipids, leptin; modulated hepatic lipid genes	Microbiota-modulating whole-food substrate (whole-food HFD model; fraction attribution not isolated)
**C. Pure-strain and boundary assays studies**				
[26]	L1/L2 glucans (L1 β-1,3/1,6-glucan-protein complex ~2200 kDa; L2 α-1,3-glucan ~2300 kDa)	Pure-strain cultivation in glucose-free MRS; donor context: NA; 9 pure probiotic strains	Microbiota/metabolites: L1/L2 stimulated *Lactobacillus* growth; L2 doubled growth rate of *Lactobacillus* Lac A; *Bifidobacterium* Bifi B grew only with *P. eryngii* extracts	Prebiotic potential (pure-strain utilization evidence)
[87]	PEP and digested products DPEP	3-stage in vitro digestion; mucus interaction for 12 h; donor context: NA	Microbiota/metabolites: DPEP interacted with extracted porcine intestinal mucus, forming an entangled network; zeta −20 to −22.03 mV; altered mucin secondary structure; host/barrier/immune: mucus interaction only; no host physiology	Digestive-fate support (upper-GI digestion and mucus-interaction evidence)

Notes: Blocks are grouped by evidence level, not ranked. APEP-A-b, a homogeneous branched β-1,6-glucan; WPEP, *Pleurotus eryngii* B-type glycosidic polysaccharide; PEP, *Pleurotus eryngii* polysaccharide; PEWS, *Pleurotus eryngii* in whole food matrix form; PEWSD, *Pleurotus eryngii* in in vitro digested form; PEWSE, *Pleurotus eryngii* form rich in β-glucans extract; DPEP, digestion product of *Pleurotus eryngii* polysaccharides. NA, not applicable. The complete set of digestion, fermentation and microbiota studies is provided in Supplementary Table S2.

## 6. Applications in Food Systems

### 6.1. Bakery, Cereal, Pasta and Staple Foods

Cereal and baked staples represent the most well-developed carrier system for *P. eryngii*. Mushroom fiber and protein fractions fit easily into these matrices, yet substitution limits are dictated by flour type, particle morphology, water-binding behavior and sensory thresholds. Two trials combined product development with gut function tests: low-dose whole-mushroom-powder-fortified durum pasta maintained processing quality and passed simulated digestion and fecal fermentation assays [89], while mushroom sourdough bread supplied fiber, β-glucans, polyphenols and vitamin D with good sensory scores and fermentative capacity [90].

Other fortified cereals and snacks (β-glucan pasta, mushroom bread, taralli, cookies, and extruded rice) gain nutritional or structural advantages at moderate addition levels; high dosages damage texture, gluten networks and sensory performance [76,91,92,93,94]. Extruded rice composites have demonstrated in vivo microbiota and metabolic effects in animal models, yet the blended matrix prevents the exclusive attribution of benefits to mushroom ingredients [76]. Mushroom incorporation into cereal foods is technically feasible, and the core unresolved formulation factor is the maximum tolerable dosage before sensory and structural degradation occurs.

### 6.2. Fermented Dairy-like and Plant-Based Systems

*P. eryngii* fractions enhance probiotic performance, texture and antioxidant properties in fermented dairy and plant-based foods, generally added at less than 1% *w*/*v*. Processing pre-treatment dominates their performance in protein-rich matrices: freeze-dried and boiled polysaccharides disperse well and boost *B. longum* in soymilk, unlike aggregated oven-dried samples [47]. Skim milk trials show they stabilize probiotics in storage and change product texture [95,96]. Polypeptide-fortified fermented sour soybean milk exerted gut immune and microbiota regulation in animal models, but soy and lactic acid bacteria prevent exclusive attribution to mushroom polypeptides [54]. Tocopherol-containing mycelial extracts only enrich yogurt’s nutrition and antioxidants [97]. These works confirm the fermentation compatibility of mushroom materials, but lack direct proof of the gut-beneficial effects of finished fermented products.

### 6.3. Meat Products and Meat Analogs

*P. eryngii* materials serve dual functions in meat products and meat analogs: as minor fortification at low doses, or core structural support at high incorporation levels. Small amounts enrich pork patties [98], and larger dosages act as fat replacers in pork sausages [99]. Moderate-to-high substitution of cuttlefish surimi with mushroom paste yields optimal texture and flavor [100]; medium freeze-dried mycelium addition boosts expansion and water retention in pea-protein analogs, though excessive inclusion causes structural collapse [101]. Mycelium and 4D-printed meat analogs even adopt mushroom biomass as the main structural matrix [32,102].

These mushroom-supplemented formulations show consistent improvements in texture, sensory performance, oxidation resistance, moisture retention and mechanical strength, demonstrating strong product development value. However, relevant gut health evaluations of final meat-based products have not yet been reported.

### 6.4. Beverages, Instant Powders and Novel Structured Foods

Beyond traditional baked, fermented and meat food matrices, *P. eryngii* can be developed into beverages, instant powders and novel structured foods, valorizing mushroom processing waste. An instant drink derived from blanching broth achieved ideal water activity and sensory performance after formulation optimization [29].

The biomass fits well with emerging manufacturing techniques: hydrocolloid-containing 3D-printing inks, printed potato snacks, in-matrix mycelial bioconversion, and thin films prepared from mycelium, stems, caps or whole mushrooms [31,103,104,105]. UVB-baked chips serve as an effective carrier for vitamin D and β-glucan enrichment [22].

Though these products demonstrate a wide processing applicability, most relevant studies merely assess processing, compositional, structural and sensory indicators, without examining the digestive and fermentative behaviors of finished foods.

### 6.5. Emulsions, Encapsulation and Delivery Systems

*P. eryngii* components function as carriers, gel matrices and impregnation supports in emulsions and encapsulation systems to encapsulate external bioactive substances. These applications center on food structure fabrication instead of direct gut health verification, extending the functional scope of mushroom materials. A soy protein–mushroom polysaccharide conjugate was fabricated as wall material for β-carotene emulsions, with its digestive and antioxidant properties evaluated using in vitro digestion and Caco-2 cell models [106]. Additional delivery formats include mushroom protein gels, structured litchi products, vacuum-impregnated stipes and fermented extracts with proven cellular antioxidative, antibacterial and anti-allergic activity [53,107,108,109]. These materials exhibit favorable encapsulation and textural functions, but no studies have assessed the colonic fermentation of finished delivery formulations.

### 6.6. From Technological Functionality to Gut-Health-Oriented Food Design

Applied food research consistently demonstrates that *P. eryngii* ingredients can be easily integrated into a wide range of food matrices, including cereal products, fermented foods, meat analogs, beverages, structured foods, and bioactive delivery systems. Incorporation of these ingredients delivers multiple functional benefits: enhanced dietary fiber and β-glucan content, improved textural properties, higher oxidative stability, and better protection of probiotic strains. Typical product applications are summarized in Table 3, with all documented formulations detailed in Supplementary Table S3.

The broad process compatibility of mushroom ingredients represents a major advantage for food product development, yet direct validation of the gut health benefits in finished products remains a critical research gap. To date, no study has applied a full analytical pipeline—combining simulated digestion, in vitro fermentation, SCFA quantification, and microbiota sequencing—to evaluate whole *P. eryngii*-containing food products. Human feeding trials have confirmed the tolerability of practical intake levels and reported improvements in selected endpoints, including glycemic responses, vitamin D status, and body composition [16,17,18,22], but none have included microbiota-related outcomes as primary endpoints. Recent studies on mushroom-fortified bread, emulsion-type meat analogs, and polysaccharide–protein coating systems have expanded the available processing technology data [110,111,112]. Moving forward, research should prioritize market-ready edible products, aligned with the established consensus that the gastrointestinal performance of dietary fiber is shaped by its chemical structure, processing history, and the surrounding food matrix [113].

A core principle for formulation design is that fermentability cannot be predicted solely from the properties of the raw starting ingredient. Food matrices that minimize severe polysaccharide depolymerization, insoluble aggregation, and excessive Maillard crosslinking generally preserve microbial accessibility to mushroom carbohydrates. In contrast, processes such as high-temperature baking, extrusion, and formulation in high-protein systems can alter mushroom carbohydrates in multiple ways—including structural protection, dilution, immobilization, or chemical transformation—depending on water activity, pH, processing intensity, and protein–polysaccharide interactions.

## 7. Key Factors Restricting Prebiotic Potential Realization of *Pleurotus eryngii* Substrates

Characteristic bioactive fractions from *P. eryngii* possess prominent prebiotic potential. However, three core factors must be addressed to convert such potential into applicable food additives, which are summarized in Table 4: cultivation and extraction workflows, substrate structure paired with feasible dosage, and the combined influence of food matrix and biological test systems.

### 7.1. Raw Material Source and Extraction Routes

Reported β-glucan contents and polysaccharide profiles of *P. eryngii* differ substantially across studies, driven by inconsistent conditions across the full production pipeline. Extraction and fractionation exert the strongest influence on the final material properties, followed by upstream cultivation conditions and then post-processing modifications. Notably, intensive post-treatments—including autohydrolysis, steam explosion, and chemical modification—can alter the polysaccharide structure to a degree comparable to extraction itself.

At the cultivation stage, the strain selection, cultivar, growth substrate, and cultivation mode collectively define the baseline levels of β-glucan, chitin, protein, and minor components across fruiting bodies, stipes, and mycelia. Extraction and purification are the single largest determinants of the final substrate composition, governing whether the recovered material is purified polysaccharide, β-glucan-enriched extract, whole-mushroom powder, oligosaccharide-rich hydrolysate, protein co-extract, or exopolysaccharide fractions. Subsequent post-treatments (drying, thermal processing, steam explosion, autohydrolysis, sulfation, and selenization) further fine-tune solubility, molecular weight distribution, molecular conformation, aggregation state, and food matrix compatibility.

Biological assay conditions introduce additional variability: the donor microbiota composition, fermentation duration, disease model, dosage, and co-occurring food components all amplify inter-study heterogeneity. This variation is not merely analytical noise; it directly modulates the substrate characteristics, microbial accessibility, and the translatability of the findings to real food systems. Full disclosure of all experimental conditions is therefore essential for valid cross-study comparisons.

Upstream cultivation conditions shape substrate performance before any extraction, drying, or formulation steps. For instance, mushrooms cultivated on wheat straw versus wheat straw–grape marc mixtures differ in both β-glucan content and fermentability in fecal cultures from older adults [20]. The fungal strain, cultivar, and tissue fraction introduce further upstream variability [19,34,35], while exopolysaccharide production in submerged culture can be modulated by adjusting the medium composition and growth parameters [30].

The extraction methodology further reshapes the polysaccharide polymer structure. Even from identical stipe biomass, sequential extraction can yield either hot-water-soluble β-1,3/1,6-glucan–protein complexes or alkali-soluble α-1,3-glucan [26]. Hot-water extraction, alkaline extraction, sequential extraction, energy-assisted extraction, autohydrolysis, and chemical modification all produce structurally distinct polysaccharide fractions; none of these approaches can be treated as interchangeable methods for generating a generic “mushroom polysaccharide” preparation.

### 7.2. Substrate Structure, Microbial Accessibility and Dosage

Molecular weight, monosaccharide composition, linkages, branching, solubility and conformation jointly govern microbial degradability, with no fixed molecular weight threshold separating active and inactive substrates. Polysaccharides ranging from 15.9 kDa to 426 kDa all induce microbiota or host regulatory effects [10,11,12,13].

Post-processing alters substrate properties without generating universally superior variants: drying methods control solubility and aggregation status [47,50]; steam explosion and autohydrolysis form unique substrate structures [46,48]; and sulfation and selenization adjust solubility and nutritional fortification effects [14,51,52]. Each treatment delivers an independent test material.

Dosage screening evaluates practical intake feasibility rather than efficacy superiority. Purified polysaccharide gavage requires sub-gram to multi-gram daily intake for adults following standard scaling rules [10,11,12,13,50,73,114,115,116], while whole-mushroom powder addition is limited by acceptable serving sizes [71,72]. Human trials of classic prebiotics and cereal β-glucans provide dosage references [117,118,119,120]. The bell-shaped response curve of APEP-A-b indicates a higher dosage does not guarantee stronger bioactivity [10].

### 7.3. Food Matrix and Biological Model

Food matrix composition determines whether mushroom fractions remain bioavailable after production. Cereals and meat analogs face sensory, oxidative and texture limitations at high substitution rates [98,99,100,101,102]; beverages and printed foods are constrained by rheology and production feasibility [31,32,103,104,105]; and emulsions, gels and fermented systems create obstacles for effect attribution during digestion assays [47,53,54,106,107,108,109]. Recent studies expand available processing techniques [110,111,112]. Notably, the substrates in final foods often differ from the initial raw ingredients.

Beyond these technical factors, the regulatory status and commercial feasibility of a given *P. eryngii* preparation also determine its ability to reach the market, both of which vary by the specific formulation and manufacturing process. In the European Union, novel food classification is governed by two core criteria: a documented history of substantial human consumption prior to 15 May 1997, and the production method used [121]. Conventional *P. eryngii* fruiting-body products with well-documented culinary use typically face lower regulatory hurdles, whereas dehydrated *P. eryngii* mycelium powder is explicitly classified as a novel food in the EU Novel Food Catalogue [122]. A separate marketing authorization covers UV-treated, vitamin D_2_-enriched mushroom powder produced from *Agaricus bisporus* (not *P. eryngii*) [123]. This approval confirms that processed mushroom ingredients are assessed for novel food status on a case-by-case basis; however, it sets only a cross-species processing precedent and does not confer any pre-established regulatory status to *P. eryngii*-derived materials. In the United States, GRAS notifications for fungal β-glucans have yielded mixed outcomes. The FDA raised no objections to β-glucans from *Ganoderma lucidum* (GRN 413) [124], whereas the evaluation of β-glucans from *Hericium erinaceus* (GRN 1124) was terminated at the notifier’s request, with no formal GRAS determination issued [125]. Accordingly, these regulatory precedents cannot be extrapolated to *P. eryngii* extracts, high-purity fractions, selenized derivatives, or chemically modified polysaccharides without ingredient-specific safety substantiation.

## 8. Conclusions and Perspectives

Current evidence indicates that well-characterized bioactive components isolated from *P. eryngii* are promising prebiotic candidates. The current limitations do not negate their prebiotic potential; research gaps primarily arise from non-uniform reporting standards and incomplete multi-layered validation. Three targeted research directions are proposed to turn early evidence into solid experimental support.

The first priority is comprehensive, unified data reporting. Full substrate information (cultivation source, strain, extraction/modification processes, molecular traits, β-glucan content, co-components and matrix type) should be recorded to separate biological differences from methodological errors. The ambiguous general label “*P. eryngii* polysaccharide” should be discarded to avoid confusion among purified fractions, extracts, powders, hydrolysates, modified derivatives, EPS and composite formulations. Standardized metadata must also be supplied for fermentation and animal tests: donor background and pooling rules, fermentation parameters, digestion protocols and SCFA metrics for fecal assays; and animal strain, gender, disease model, administration scheme and BSA-based HED conversion rationale for in vivo trials [114,115,116].

Second, functional assessment should target marketable finished mushroom-fortified foods rather than isolated ingredients. The integrated testing pipeline covers ingredient characterization, post-processing re-evaluation, simulated digestion of final products, fecal fermentation of digesta, metabolite quantification and microbiota sequencing. Human tolerance and efficacy assessments can proceed only after favorable gut-modulating effects are verified for complete food matrices.

Third, conclusive validation demands a pilot human trial adopting fully defined *P. eryngii* substrates and microbiota-focused endpoints. Whole-mushroom powder fits food compatibility tests, whereas purified or β-glucan-rich extracts support dosage tolerance and mechanistic research. Trial results become more comparable when matched with mushroom and cereal β-glucan reference groups under identical experimental workflows.

These three suggestions do not challenge the promising prebiotic potential of *P. eryngii* substrates. Instead, they form a clear research roadmap to achieve full validation of these promising prebiotic candidates.

## Figures and Tables

**Table 3:** Representative *P. eryngii*-based food products by category.

Validation Status	Ref.	Food System	*P. eryngii* Ingredient/Level	Processing/Design	Main Outcomes	Validation Context
**A. Bakery, cereal, pasta and staple foods**						
P-Ferm	[84]	Fresh durum wheat semolina pasta	Whole powder, 8.62% and 17.24%	Pasteurized prototype in modified atmosphere; 4 °C storage	8.62%: fiber 8.7%, cooking loss 5.4%, acceptable sensory; shelf-life stable 110 d	Food composite; gut-related readout is culture-plating-based
P-Cell	[88]	Taralli baked snack	Whole powder, 5% and 10%	Dough rested 4 °C 15 min; baked 200 °C 20 min	10%: 7.91% dietary fiber, 3.03 g/100 g β-glucan; sensory acceptance comparable despite bitterness/astringency	Cell and inflammation-related readouts; microbiota profiling remains a next step
**B. Fermented dairy-like and plant-based systems**						
P-Pro	[47]	Soymilk matrix	PEPS from differently heat-treated mushrooms; 0.5% *w*/*v*	Added to soy protein isolate-based soymilk	Promoted *B. longum* growth; FDPEPS/BTPEPS soluble, ODPEPS formed aggregates	Probiotic-growth support in formulation
P-Animal	[54]	Fermented sour soybean milk	<3 kDa polypeptides; 0.3/0.5/0.7% groups	Soy milk + lactose/sucrose/gelatin/β-cyclodextrin + mixed LAB; 42 °C, 5.5 h	FPEP LAB count 4.38 × 10^8^ CFU/mL vs. 1.84 × 10^8^ SSM; pH 4.26; WHC 53.56%	Composite fermented soymilk system; attribution to the polypeptide component remains limited
**C. Meat products and plant-based meat analogs**						
T	[96]	Pea protein low-moisture meat analog	Freeze-dried mycelium, 0–40% *w*/*w*	Twin-screw extrusion at 140 °C	30% mycelium improved WHC and expansion; 40% caused structural disintegration	Texture and formulation evidence for meat analog development
**D. Beverages, instant powders and novel structured foods**						
T	[22]	Mushroom chips/snack	Sliced mushroom; UVB-irradiated and baked	Baked 120 °C 20 min; UVB 120 min; seasoning variants	25.43 g/100 g protein; 8.5 g/100 g crude fiber; 32.18% β-glucans; vitamin D_2_ increased	Snack nutrition and sensory evidence
**E. Emulsions, encapsulation and delivery systems**						
P-Dig	[101]	β-carotene emulsion/delivery system	SPI–PEP conjugate; β-carotene-loaded emulsion	High-pressure microfluidization at 90 MPa	Encapsulation efficiency 65.13%; stable pH 4.0–11.0; intestinal FFA release 89.73%	Delivery-system digestion evidence; colonic fermentability of the *P. eryngii* component remains untested

Notes: Validation status refers to conclusion generated with the final formulated food. Codes: T, technological/nutritional characterization; P-Ferm, partial culture-based fermentation; P-Dig, partial simulated digestion; P-Cell, partial cell, barrier or inflammation-related readouts; P-Animal, partial animal-model microbiota readouts after product consumption; P-Pro, partial probiotic growth/survival or simulated-GI tolerance. PEPS, *Pleurotus eryngii* polysaccharide; FDPEPS, freeze-dried form of *Pleurotus eryngi* polysaccharides; BTPEPS, boiling-treated form of *Pleurotus eryngi* polysaccharides; ODPEPS, oven-dried *Pleurotus eryngi* polysaccharides; FPEP, fermented with PEP (*Pleurotus eryngii* polypeptides with a molecular weight of <3 kDa). The complete product-level inventory is provided in Supplementary Table S3.

**Table 4:** Key factors influencing prebiotic potential and food-matrix performance.

Design Factor	Range of Variation	Effect Magnitude/Qualitative Direction	Supporting Studies	Key Example Ref.	Practical Implication	Translation Note
Cultivation substrate, strain and growth mode	Solid-substrate cultivation on commercial or agricultural by-product substrates; submerged-culture mycelial fermentation; strain and varietal variation.	Within-study β-glucan content differed between wheat-straw and wheat-straw/grape-marc cultivation (38.7% vs. 42.2%) [20]; varietal β-glucan differences (22.41% vs. 9.51%) [19], within-variety PELPS fraction diversity [35] and submerged-culture EPS production [30] were also reported.	6 entries	[19,20,30,33,34,35]	Cultivation substrate and strain selection are key upstream determinants of β-glucan content and of the microbiota responses being tested.	Independent replication and matched fruiting-body versus submerged-culture comparisons will clarify how general these upstream effects are.
Extraction and fractionation route	Hot-water, cold-water, alkali, salt, enzyme-assisted, ultrasound, microwave, steam-explosion and autohydrolysis routes; single-step versus sequential extraction.	Extraction route determines whether the recovered material is a purified polysaccharide, protein co-extract, hydrolysate or composite; one sequential protocol resolved five structural types from a single source.	>20 entries	[26,42,45,46,49]	Extraction route is the key determinant of substrate-class identity and should be reported alongside structural data.	Matched cross-route comparisons will clarify how extraction and fractionation shape substrate-specific outcomes.
Drying, processing and chemical modification	Freeze-, oven-, boiling- and spray-drying; steam explosion; autohydrolysis; sulfation and selenization.	Spray-drying altered particle morphology and chain length; oven-drying generated insoluble Maillard aggregates; sulfation increased solubility; selenization preserved the polysaccharide backbone with a small Mw decrease.	10 entries	[14,46,47,48,50,51,52]	Drying and modification should be treated as formation steps that alter food-matrix compatibility and accessibility.	Modified derivatives and Maillard aggregates should be interpreted as formation products with their own substrate identities.
Molecular structure and accessibility	Mw from low single-digit kDa to megadalton-scale fractions; β- and α-linked glucans, galactans and mannans; triple-helix and rigid-rod conformations.	Gut-related readouts occur across the surveyed Mw range, so Mw alone does not predict activity; linkage, branching and conformation remain necessary descriptors.	10 entries	[10,11,12,13,42,55]	Substrates should be defined by Mw, monosaccharide profile, linkage, branching and conformation rather than by species name alone.	Matched-protocol comparisons will strengthen conformation–microbiota links across substrate classes.
Dose and formulation level	Mouse gavage 100–1200 mg/kg/day; dietary inclusion 1.5–3.0% *w*/*w*; in vitro fermentation 1 g/vessel or 2% *w*/*v*; food-matrix addition from <1% to >20%.	Gavage-derived HEDs fall in the sub-gram to several-gram/day range; whole-powder inclusion is assumption-dependent (≈11.7–14.6 g/day at 1.5% *w*/*w*; ≈29.3 g/day at 3.0% *w*/*w*). A bell-shaped response was reported in one APEP-A-b study.	12 entries	[10,11,13,28,50,70,71]	HED conversion provides dose-feasibility anchors that must be matched to realistic formulation levels.	HED supports dose-feasibility assessment; efficacy requires human testing, and non-monotonic dose response should be checked in replication studies.
Food matrix interaction	Cereal, fermented dairy-like, meat, beverage, structured-food, emulsion and delivery matrices; thermal processing, extrusion, fermentation, encapsulation and bioconversion.	Cereal matrices tolerate higher inclusion levels than low-level meat applications; final-food validation remains absent across Table 3.	29 food-application studies (Table S3)	[32,47,76,89,93,106]	Product development should distinguish technological/nutritional functionality from gut-health-oriented validation.	Product-level validation is the next step for finished-food applications, especially when digestion, fermentation, metabolite and microbiota readouts are combined.
Biological model and donor variability	Pure-strain assays; human fecal fermentation with young adult, elderly and pediatric donors; rodent and poultry models; disease states and toxicity challenges.	Donor population, model construction and fermentation duration strongly condition microbiota and SCFA outcomes.	Pooled across Table 2	[11,13,20,28,33,38,54,62,66]	Standardized donor reporting and matched protocols are needed for cross-study comparability.	Matched donor and model reporting will clarify substrate-specific effects across study systems.

Notes: PELPS, water-soluble glucans in *Pleurotus eryngii*; APEP-A-b, a homogeneous branched β-1,6-glucan; HED, human-equivalent dose; Mw, molecular weight.

## Data Availability

No new data were created or analyzed in this study. Data sharing is not applicable to this article.

## Supplementary Materials

The following supporting information can be downloaded at https://www.mdpi.com/article/10.3390/foods15142527/s1, Literature search and study selection; Table S1: Complete inventory of formation routes and structural features of *P. eryngii*-derived candidate prebiotic substrates; Table S2: Complete inventory of digestive-fate, fermentation and microbiota-related studies of *P. eryngii*-derived substrates, grouped by evidence model; Table S3: Complete product-level inventory of *P. eryngii*-based food applications, grouped by food category and annotated with validation status.

## Abbreviations

The following abbreviations are used in this manuscript:

AFB1	Aflatoxin B1
AUC	Area under the curve
BCAA	Branched-chain amino acid
BSA	Body surface area
CFU	Colony-forming unit
DAI	Disease activity index
DEAE	Diethylaminoethyl (anion-exchange chromatography)
DS	Degree of substitution
DSS	Dextran sulfate sodium
EPS	Exopolysaccharide
FFA	Free fatty acid
FT-IR	Fourier-transform infrared spectroscopy
GABA	γ-Aminobutyric acid
GI	Gastrointestinal
GPR43	G-protein-coupled receptor 43 (free fatty acid receptor 2)
HED	Human-equivalent dose
HFD	High-fat diet
HRP	Horseradish peroxidase
IDF	Insoluble dietary fiber
IL	Interleukin (e.g., IL-1β, IL-6, and IL-10)
INFOGEST	International standardized static in vitro digestion protocol
LAB	Lactic acid bacteria
LPS	Lipopolysaccharide
MUC2	Mucin 2
Mw	Molecular weight
NF-κB	Nuclear factor kappa B
NMR	Nuclear magnetic resonance
OGTT	Oral glucose tolerance test
pGI	Predicted (in vitro) glycemic index
SCFA	Short-chain fatty acid
sIgA	Secretory immunoglobulin A
SPI	Soy protein isolate
STZ	Streptozotocin
TBARS	Thiobarbituric acid reactive substance
TLR4	Toll-like receptor 4
TNF-α	Tumor necrosis factor alpha
VFA	Volatile fatty acid
WHC	Water-holding capacity
ZO-1	Zonula occludens-1 (tight-junction protein)

## References

[1] Gibson G.R., Roberfroid M.B. (1995). Dietary Modulation of the Human Colonic Microbiota: Introducing the Concept of Prebiotics. J. Nutr..

[2] Gibson G.R., Hutkins R., Sanders M.E., Prescott S.L., Reimer R.A., Salminen S.J., Scott K., Stanton C., Swanson K.S., Cani P.D. (2017). Expert consensus document: The International Scientific Association for Probiotics and Prebiotics (ISAPP) consensus statement on the definition and scope of prebiotics. Nat. Rev. Gastroenterol. Hepatol..

[3] Hutkins R.W., Krumbeck J.A., Bindels L.B., Cani P.D., Fahey G., Goh Y.J., Hamaker B., Martens E.C., Mills D.A., Rastall R.A. (2016). Prebiotics: Why definitions matter. Curr. Opin. Biotechnol..

[4] Bindels L.B., Delzenne N.M., Cani P.D., Walter J. (2015). Towards a more comprehensive concept for prebiotics. Nat. Rev. Gastroenterol. Hepatol..

[5] Brodkorb A., Egger L., Alminger M., Alvito P., Assunção R., Ballance S., Bohn T., Bourlieu-Lacanal C., Boutrou R., Carrière F. (2019). INFOGEST static in vitro simulation of gastrointestinal food digestion. Nat. Protoc..

[6] Sonnenburg E.D., Sonnenburg J.L. (2014). Starving our microbial self: The deleterious consequences of a diet deficient in microbiota-accessible carbohydrates. Cell Metab..

[7] Makki K., Deehan E.C., Walter J., Bäckhed F. (2018). The impact of dietary fiber on gut microbiota in host health and disease. Cell Host Microbe.

[8] Cantu-Jungles T.M., Hamaker B.R. (2020). New view on dietary fiber selection for predictable shifts in gut microbiota. mBio.

[9] Mattioli L.B., Camarda L., Aicardi M., Pasquali E., Corazza I., Budriesi R. (2026). Edible Mushrooms as Emerging Prebiotic Sources: Gut Microbiota Modulation and SCFA-Mediated Health Effects. Foods.

[10] Wang X., Qu Y., Wang Y., Wang X., Xu J., Zhao H., Zheng D., Sun L., Tai G., Zhou Y. (2022). β-1,6-Glucan from *Pleurotus eryngii* Modulates the Immunity and Gut Microbiota. Front. Immunol..

[11] Li L., Sun J., Zhou J., Song P., Zuo S., Chang Z., Chen G., Guo J., Diao X., Jiang X. (2025). Structural characteristics of *Pleurotus eryngii* polysaccharide and its protective mechanisms against mastitis in mice. Int. J. Biol. Macromol..

[12] Ma G., Kimatu B.M., Zhao L., Yang W., Pei F., Hu Q. (2017). In vivo fermentation of a *Pleurotus eryngii* polysaccharide and its effects on fecal microbiota composition and immune response. Food Funct..

[13] Ma G., Hu Q., Han Y., Du H., Yang W., Pan C., Cao X., Muinde Kimatu B., Pei F., Xiao H. (2021). Inhibitory effects of β-type glycosidic polysaccharide from *Pleurotus eryngii* on dextran sodium sulfate-induced colitis in mice. Food Funct..

[14] Yuan M., Huang X., Tian S., Ma S., Guo Y., Tao M. (2024). Selenized modification and structural characterization of *Pleurotus eryngii* polysaccharides and their immunomodulatory activity. Process Biochem..

[15] Ji Y., Hu Q., Zhang X., Ma G., Zhao R., Zhao L. (2024). Effects of selenium biofortification on *Pleurotus eryngii* protein structure and digestive properties and its mitigation of lead toxicity: An in vitro and in vivo study. Food Chem..

[16] Kleftaki S.-A., Simati S., Amerikanou C., Gioxari A., Tzavara C., Zervakis G.I., Kalogeropoulos N., Kokkinos A., Kaliora A.C. (2022). *Pleurotus eryngii* improves postprandial glycaemia, hunger and fullness perception, and enhances ghrelin suppression in people with metabolically unhealthy obesity. Pharmacol. Res..

[17] Kleftaki S.-A., Amerikanou C., Gioxari A., Lantzouraki D.Z., Sotiroudis G., Tsiantas K., Tsiaka T., Tagkouli D., Tzavara C., Lachouvaris L. (2022). A Randomized Controlled Trial on *Pleurotus eryngii* Mushrooms with Antioxidant Compounds and Vitamin D_2_ in Managing Metabolic Disorders. Antioxidants.

[18] Amerikanou C., Kleftaki S.-A., Gioxari A., Tagkouli D., Kasoura A., Simati S., Tzavara C., Kokkinos A., Kalogeropoulos N., Kaliora A.C. (2025). The Performance of *Pleurotus eryngii* β-Glucans on Protein Digestion and the Release of Free Amino Acids in the Bloodstream of Obese Adults. Foods.

[19] Jaffali C., Synytsya A., Bleha R., Khadhri A., Aschi-Smiti S., Smrčková P., Klouček P. (2024). Characterization of two Tunisian strains of culinary oyster mushroom *Pleurotus eryngii*: Differences in the biopolymer composition. J. Food Compos. Anal..

[20] Mitsou E.K., Saxami G., Stamoulou E., Kerezoudi E., Terzi E., Koutrotsios G., Bekiaris G., Zervakis G.I., Mountzouris K.C., Pletsa V. (2020). Effects of Rich in β-Glucans Edible Mushrooms on Aging Gut Microbiota Characteristics: An In Vitro Study. Molecules.

[21] Afonso T.B., Marçal S., Vale P., Sousa A.S., Nunes J., Pintado M. (2025). Exploring the Bioactive Potential of Mushroom Aqueous Extracts: Antimicrobial, Antioxidant, and Prebiotic Properties. Appl. Sci..

[22] Amerikanou C., Tagkouli D., Tsiaka T., Lantzouraki D.Z., Karavoltsos S., Sakellari A., Kleftaki S.-A., Koutrotsios G., Giannou V., Zervakis G.I. (2023). *Pleurotus eryngii* Chips—Chemical Characterization and Nutritional Value of an Innovative Healthy Snack. Foods.

[23] Synytsya A., Novák M. (2013). Structural diversity of fungal glucans. Carbohydr. Polym..

[24] Rop O., Mlcek J., Jurikova T. (2009). Beta-glucans in higher fungi and their health effects. Nutr. Rev..

[25] Ruthes A.C., Smiderle F.R., Iacomini M. (2015). D-Glucans from edible mushrooms: A review on the extraction, purification and chemical characterization approaches. Carbohydr. Polym..

[26] Synytsya A., Míčková K., Synytsya A., Jablonský I., Spěváček J., Erban V., Kováříková E., Čopíková J. (2009). Glucans from fruit bodies of cultivated mushrooms *Pleurotus ostreatus* and *Pleurotus eryngii*: Structure and potential prebiotic activity. Carbohydr. Polym..

[27] Zheng H.-G., Chen J.-C., Weng M.-J., Ahmad I., Zhou C.-Q. (2020). Structural characterization and bioactivities of a polysaccharide from the stalk residue of *Pleurotus eryngii*. Food Sci. Technol..

[28] Fu Y., Wang Q., Guo Y., Koci M., Lu Z., Zeng X., Wang Y., Tang Y., Ma Q., Ji C. (2024). *Pleurotus eryngii* polysaccharides alleviate aflatoxin B1-induced liver inflammation in ducks involving in remodeling gut microbiota and regulating SCFAs transport via the gut-liver axis. Int. J. Biol. Macromol..

[29] Lin S.-D., Wu Y.-T., Lo Y.-C., Mau J.-L. (2018). Quality characteristics of centrifuged broth from blanched *Pleurotus eryngii* and its application as instant drink. J. Food Process. Preserv..

[30] Jing X., Mao D., Geng L., Xu C. (2013). Medium optimization, molecular characterization, and bioactivity of exopolysaccharides from *Pleurotus eryngii*. Arch. Microbiol..

[31] Li H., Xie S., Cao S., Hu L., Xu D., Zhang J., Mo H., Liu Z. (2022). Bioconversion of High-Calorie Potato Starch to Low-Calorie β-Glucan via 3D Printing Using *Pleurotus eryngii* Mycelia. Foods.

[32] Hu X., Tang H., Wang J., Zou L., Su X., Xu B. (2026). Mycelial growth stimulus-responsive 4D printing: A novel approach for mycelium-based meat analogs fabrication. Innov. Food Sci. Emerg. Technol..

[33] Kerezoudi E.N., Vlassopoulou M., Mitsou E.K., Saxami G., Koutrotsios G., Taflampa I., Mountzouris K.C., Rangel I., Brummer R.J., Zervakis G.I. (2025). In vitro fermentation of whole matrix, digested products and β-glucan enriched extract of *Pleurotus eryngii* mushrooms distinctively impact the fecal microbiota of healthy older adults. Hum. Nutr. Metab..

[34] Jaffali C., Synytsya A., Khadhri A., Aschi-Smiti S., Bleha R., Jozífek M., Kvasnička F., Klouček P. (2025). Structure and strain specificity for polysaccharides from king oyster mushroom (*Pleurotus eryngii*) fruiting bodies. Int. J. Biol. Macromol..

[35] Cateni F., Zacchigna M., Procida G., Venturella G., Ferraro V., Gargano M.L. (2020). Polysaccharides from *Pleurotus eryngii* var. *elaeoselini* (Agaricomycetes), a New Potential Culinary-Medicinal Oyster Mushroom from Italy. Int. J. Med. Mushrooms.

[36] Han X., Yang D., Zhang S., Liu X., Zhao Y., Song C., Sun Q. (2023). Characterization of insoluble dietary fiber from *Pleurotus eryngii* and evaluation of its effects on obesity-preventing or relieving effects via modulation of gut microbiota. J. Future Foods.

[37] Zhao Y., Chen X., Jia W., Gong G., Zhao Y., Li G., Zhou J., Li X., Zhao Y., Ma W. (2020). Extraction, isolation, characterisation, antioxidant and anti-fatigue activities of *Pleurotus eryngii* polysaccharides. Int. J. Food Sci. Technol..

[38] Ma G., Xu Q., Du H., Muinde Kimatu B., Su A., Yang W., Hu Q., Xiao H. (2022). Characterization of polysaccharide from *Pleurotus eryngii* during simulated gastrointestinal digestion and fermentation. Food Chem..

[39] Ren D., Wang N., Guo J., Yuan L., Yang X. (2016). Chemical characterization of *Pleurotus eryngii* polysaccharide and its tumor-inhibitory effects against human hepatoblastoma HepG-2 cells. Carbohydr. Polym..

[40] Yan J., Meng Y., Zhang M., Zhou X., Cheng H., Sun L., Zhou Y. (2019). A 3-O-methylated heterogalactan from *Pleurotus eryngii* activates macrophages. Carbohydr. Polym..

[41] Hao C., Yang J., Liang T., Zhang J., Sun R. (2017). Structural elucidation and morphological observation of a polysaccharide from *Pleurotus eryngii* obtained by alkaline extraction. J. Carbohydr. Chem..

[42] Abreu H., Zavadinack M., Smiderle F.R., Cipriani T.R., Cordeiro L.M.C., Iacomini M. (2021). Polysaccharides from *Pleurotus eryngii*: Selective extraction methodologies and their modulatory effects on THP-1 macrophages. Carbohydr. Polym..

[43] Liu X., Wang L., Zhang C., Wang H., Zhang X., Li Y. (2015). Structure characterization and antitumor activity of a polysaccharide from the alkaline extract of king oyster mushroom. Carbohydr. Polym..

[44] Wang H., Ma S., Mariga A.M., Hu Q., Xu Q., Su A., Ma N., Ma G. (2024). Structural characterization and anti-inflammatory activities of novel polysaccharides obtained from *Pleurotus eryngii*. Food Sci. Hum. Wellness.

[45] Li X., Tao Q., Hu Q., Ma N., Ma G. (2024). In vitro gastrointestinal digestion and fecal fermentation of *Pleurotus eryngii* proteins extracted using different methods: Insights for the utilization of edible mushroom-based proteins as novel nutritional and functional components. Food Funct..

[46] Rodríguez-Seoane P., Díaz-Reinoso B., González-Muñoz M.J., Fernández De Ana Portela C., Domínguez H. (2019). Innovative technologies for the extraction of saccharidic and phenolic fractions from *Pleurotus eryngii*. LWT.

[47] Li S., Shah N.P. (2016). Characterization, antioxidative and bifidogenic effects of polysaccharides from *Pleurotus eryngii* after heat treatments. Food Chem..

[48] Qiu J., Zheng P., Dai W., Zheng Z., Lin X., Hu J., Zeng S., Lin S. (2024). Steam Explosion-Assisted Extraction of Polysaccharides from *Pleurotus eryngii* and Its Influence on Structural Characteristics and Antioxidant Activity. Foods.

[49] Sun Z., Song Z., Zhou A., Lin Y., Yao J., Mayo K.H., Zhou Y., Sun L. (2026). Polysaccharides from *Pleurotus eryngii*: Sequential extraction and structural characterization. Food Chem. X.

[50] Chen J., Zhou M., Chen L., Yang C., Deng Y., Li J., Sun S. (2024). Evaluation of Physicochemical Properties and Prebiotics Function of a Bioactive *Pleurotus eryngii* Aqueous Extract Powder Obtained by Spray Drying. Nutrients.

[51] Jung H.Y., Bae I.Y., Lee S., Lee H.G. (2011). Effect of the degree of sulfation on the physicochemical and biological properties of *Pleurotus eryngii* polysaccharides. Food Hydrocoll..

[52] Li S., Shah N.P. (2014). Antioxidant and antibacterial activities of sulphated polysaccharides from *Pleurotus eryngii* and *Streptococcus thermophilus* ASCC 1275. Food Chem..

[53] Kim D., Ko Y.H., Chung H.C., Han G.D. (2015). Straightforward bacterial-fungal fermentation between *Lactobacillus plantarum* and *Pleurotus eryngii* for synergistic improvement of bioactivity. Food Sci. Biotechnol..

[54] Song X., Xu X., Chen W. (2022). Antioxidant and Immunostimulatory Activities of Fermented Sour Soybean Milk Added with Polypeptides from *Pleurotus eryngii*. Front. Microbiol..

[55] Carbonero E.R., Gracher A.H.P., Smiderle F.R., Rosado F.R., Sassaki G.L., Gorin P.A.J., Iacomini M. (2006). A β-glucan from the fruit bodies of edible mushrooms *Pleurotus eryngii* and *Pleurotus ostreatoroseus*. Carbohydr. Polym..

[56] Flint H.J., Scott K.P., Duncan S.H., Louis P., Forano E. (2012). Microbial degradation of complex carbohydrates in the gut. Gut Microbes.

[57] Du B., Meenu M., Liu H., Xu B. (2019). A Concise Review on the Molecular Structure and Function Relationship of β-Glucan. Int. J. Mol. Sci..

[58] Minekus M., Alminger M., Alvito P., Ballance S., Bohn T., Bourlieu C., Carrière F., Boutrou R., Corredig M., Dupont D. (2014). A standardised static in vitro digestion method suitable for food—An international consensus. Food Funct..

[59] Ma S., Duan Y., Yu Y., Hu Q., Tao Q., Li X., Kimatu B.M., Ma G. (2025). Effects and Mechanisms of *Pleurotus eryngii* Polysaccharide on Intestinal Barrier Damage: Based on the Perspective of Its Interaction with Intestinal Mucus during Gut Digestion. J. Agric. Food Chem..

[60] Ma G., Ma S., Du H., Li X., Tao Q., Hu Q., Xiao H. (2024). Interactions between intestinal microbial fermentation products of *Pleurotus eryngii* polysaccharide with gut mucus. Food Funct..

[61] Tao Q., Li X., Duan Y., Yu Y., Hu Q., Ma N., Su A., Yang C., Ma G. (2026). Reveal the potential key role of characteristic metabolites of wet glycosylated *Pleurotus eryngii* protein-Oat β-glucan complex in the process of improving intestinal barrier function-from the perspective of intestinal digestion and microbial fermentation. Food Biosci..

[62] Tao Q., Li X., Ma S., Hu Q., Ma G. (2025). A comparison study on compounding oat β-glucan onto *Pleurotus eryngii* protein by using different methods: In vitro digestion and fermentation characteristics. Food Hydrocoll..

[63] Tsai S.-Y., So H.-Y., Lin C.-P. (2026). Assessing the prebiotic effects on microorganism growth through various irradiation treatments of *Pleurotus eryngii* polysaccharides using microcalorimetry. J. Therm. Anal. Calorim..

[64] Zhao R., Yang W., Pei F., Zhao L., Hu Q. (2018). In vitro fermentation of six kinds of edible mushrooms and its effects on fecal microbiota composition. LWT.

[65] Ji Y., Hu Q., Ma G., Yu A., Zhao L., Zhang X., Zhao R. (2022). Selenium biofortification in *Pleurotus eryngii* and its effect on lead adsorption of gut microbiota via in vitro fermentation. Food Chem..

[66] Christodoulou P., Vlassopoulou M., Zervou M., Xanthakos E., Moulos P., Koutrotsios G., Zervakis G.I., Kerezoudi E.N., Mitsou E.K., Saxami G. (2023). In Vitro Fermentation of *Pleurotus eryngii* Mushrooms by Human Fecal Microbiota: Metataxonomic Analysis and Metabolomic Profiling of Fermentation Products. J. Fungi.

[67] Boulaka A., Mantellou P., Stanc G.-M., Souka E., Valavanis C., Saxami G., Mitsou E., Koutrotsios G., Zervakis G.I., Kyriacou A. (2022). Genoprotective activity of the *Pleurotus eryngii* mushrooms following their in vitro and in vivo fermentation by fecal microbiota. Front. Nutr..

[68] Saxami G., Mitsou E.K., Kerezoudi E.N., Mavrouli I., Vlassopoulou M., Koutrotsios G., Mountzouris K.C., Zervakis G.I., Kyriacou A. (2023). In Vitro Fermentation of Edible Mushrooms: Effects on Faecal Microbiota Characteristics of Autistic and Neurotypical Children. Microorganisms.

[69] Koh A., De Vadder F., Kovatcheva-Datchary P., Bäckhed F. (2016). From Dietary Fiber to Host Physiology: Short-Chain Fatty Acids as Key Bacterial Metabolites. Cell.

[70] Parada Venegas D., De la Fuente M.K., Landskron G., González M.J., Quera R., Dijkstra G., Harmsen H.J.M., Faber K.N., Hermoso M.A. (2019). Short chain fatty acids (SCFAs)-mediated gut epithelial and immune regulation and its relevance for inflammatory bowel diseases. Front. Immunol..

[71] Hu Q., Yuan B., Wu X., Du H., Gu M., Han Y., Yang W., Song M., Xiao H. (2019). Dietary Intake of *Pleurotus eryngii* Ameliorated Dextran-Sodium-Sulfate-Induced Colitis in Mice. Mol. Nutr. Food Res..

[72] Du H., Han Y., Ma G., Tan C., Hu Q., Xiao H. (2024). Dietary intake of whole king oyster mushroom (*Pleurotus eryngii*) attenuated obesity via ameliorating lipid metabolism and alleviating gut microbiota dysbiosis. Food Res. Int..

[73] Duan Y., Yu Y., Pan H., Bao R., Zhang R., Hu Q., Ma G. (2025). Novel anti-obesity mechanisms of *Pleurotus eryngii*: Based on the regulatory effects of potential key gut microbiota and associated metabolites. Food Biosci..

[74] Furusawa Y., Obata Y., Fukuda S., Endo T.A., Nakato G., Takahashi D., Nakanishi Y., Uetake C., Kato K., Kato T. (2013). Commensal microbe-derived butyrate induces the differentiation of colonic regulatory T cells. Nature.

[75] Mann E.R., Lam Y.K., Uhlig H.H. (2024). Short-chain fatty acids: Linking diet, the microbiome and immunity. Nat. Rev. Immunol..

[76] Zhou L., Jiang W., Pei F., Ji Y., Su A., Ma G., Zhang Y., Hu Q., Ma N. (2025). *Pleurotus eryngii* extruded rice improved HFD/STZ-induced type 2 diabetic mice by activating PI3K/AKT signaling pathway and regulating gut microbiota. J. Funct. Foods.

[77] Wang J., Liu X., Jin D., Wang M., Zhang N., Zhao Y., Yang W., Li N., Chen X., Gong P. (2026). Immunomodulatory mechanism of *Pleurotus eryngii* polysaccharides in acrylamide-induced mice via the P2X7-NLRP3 pathway, metabolic homeostasis and gut microbiota. Food Biosci..

[78] Nakahara D., Nan C., Mori K., Hanayama M., Kikuchi H., Hirai S., Egashira Y. (2020). Effect of mushroom polysaccharides from *Pleurotus eryngii* on obesity and gut microbiota in mice fed a high-fat diet. Eur. J. Nutr..

[79] Li X., Tao Q., Hu Q., Hou T., Yu Y., Duan Y., Ma G. (2025). Positive effect of a novel protein from *Pleurotus eryngii* on improving colitis: Exploring potential mechanisms from the perspective of intestinal metabolism. Food Funct..

[80] Derrien M., Belzer C., de Vos W.M. (2017). *Akkermansia muciniphila* and its role in regulating host functions. Microb. Pathog..

[81] Miquel S., Martín R., Rossi O., Bermúdez-Humarán L.G., Chatel J.M., Sokol H., Thomas M., Wells J.M., Langella P. (2013). *Faecalibacterium prausnitzii* and human intestinal health. Curr. Opin. Microbiol..

[82] Depommier C., Everard A., Druart C., Plovier H., Van Hul M., Vieira-Silva S., Falony G., Raes J., Maiter D., Delzenne N.M. (2019). Supplementation with *Akkermansia muciniphila* in overweight and obese human volunteers: A proof-of-concept exploratory study. Nat. Med..

[83] Magne F., Gotteland M., Gauthier L., Zazueta A., Pesoa S., Navarrete P., Balamurugan R. (2020). The Firmicutes/Bacteroidetes Ratio: A Relevant Marker of Gut Dysbiosis in Obese Patients?. Nutrients.

[84] Saxami G., Kerezoudi E.N., Mitsou E.K., Koutrotsios G., Zervakis G.I., Pletsa V., Kyriacou A. (2021). Fermentation Supernatants of *Pleurotus eryngii* Mushroom Ameliorate Intestinal Epithelial Barrier Dysfunction in Lipopolysaccharide-Induced Caco-2 Cells via Upregulation of Tight Junctions. Microorganisms.

[85] Kerezoudi E.N., Saxami G., Zervakis G.I., Pletsa V., Brummer R.J., Kyriacou A., Rangel I. (2025). Effects of In Vitro Fermented *Pleurotus eryngii* on Intestinal Barrier Integrity and Immunomodulation in a Lipopolysaccharide-Induced Colonic Model. Biomedicines.

[86] Kerezoudi E.N., Zervakis G.I., Pletsa V., Kyriacou A., Brummer R.J., Rangel I. (2025). *Pleurotus eryngii* Mushrooms Fermented with Human Fecal Microbiota Protect Intestinal Barrier Integrity: Immune Modulation and Signalling Pathways Counter Deoxycholic Acid-Induced Disruption in Healthy Colonic Tissue. Nutrients.

[87] Ma S., Li X., Tao Q., Hu Q., Yang W., Kimatu B.M., Ma G. (2024). The effect of in vitro digestion on the interaction between polysaccharides derived from *Pleurotus eryngii* and intestinal mucus. Food Sci. Nutr..

[88] Vlassopoulou M., Paschalidis N., Savvides A.L., Saxami G., Mitsou E.K., Kerezoudi E.N., Koutrotsios G., Zervakis G.I., Georgiadis P., Kyriacou A. (2022). Immunomodulating Activity of *Pleurotus eryngii* Mushrooms Following Their In Vitro Fermentation by Human Fecal Microbiota. J. Fungi.

[89] Calasso M., Lisi A., Ressa A., Caponio G.R., Difonzo G., Minervini F., Gargano M.L., Vacca M., De Angelis M. (2025). Incorporating Fresh Durum Wheat Semolina Pasta Fortified with Cardoncello (*Pleurotus eryngii*) Mushroom Powder as a Mediterranean Diet Staple. Antioxidants.

[90] Cirlincione F., Venturella G., Gargano M.L., Ferraro V., Gaglio R., Francesca N., Rizzo B.A., Russo G., Moschetti G., Settanni L. (2022). Functional bread supplemented with *Pleurotus eryngii* powder: A potential new food for human health. Int. J. Gastron. Food Sci..

[91] Kim S., Lee J.-W., Heo Y., Moon B. (2016). Effect of *Pleurotus eryngii* Mushroom β-Glucan on Quality Characteristics of Common Wheat Pasta. J. Food Sci..

[92] Gaglio R., Guarcello R., Venturella G., Palazzolo E., Francesca N., Moschetti G., Settanni L., Saporita P., Gargano M.L. (2019). Microbiological, chemical and sensory aspects of bread supplemented with different percentages of the culinary mushroom *Pleurotus eryngii* in powder form. Int. J. Food Sci. Technol..

[93] Caponio G.R., Difonzo G., Troilo M., Marcotuli I., Gadaleta A., Tamma G., Gargano M.L., Cirlincione F. (2025). Enhancing the Nutritional and Health-Related Properties of Taralli Through the Use of *Pleurotus eryngii*: Focus on Antioxidant and Anti-Inflammatory Properties. Antioxidants.

[94] Biao Y., Chen X., Wang S., Chen G., McClements D.J., Zhao L. (2020). Impact of mushroom (*Pleurotus eryngii*) flour upon quality attributes of wheat dough and functional cookies-baked products. Food Sci. Nutr..

[95] Li S., Shah N.P. (2015). Effects of *Pleurotus eryngii* polysaccharides on bacterial growth, texture properties, proteolytic capacity, and angiotensin-I-converting enzyme–inhibitory activities of fermented milk. J. Dairy Sci..

[96] Chou W.-T., Sheih I.-C., Fang T.J. (2013). The Applications of Polysaccharides from Various Mushroom Wastes as Prebiotics in Different Systems. J. Food Sci..

[97] Bouzgarrou C., Amara K., Reis F.S., Barreira J.C.M., Skhiri F., Chatti N., Martins A., Barros L., Ferreira I.C.F.R. (2018). Incorporation of tocopherol-rich extracts from mushroom mycelia into yogurt. Food Funct..

[98] Zhang M., Chai Y., Li F., Bao Y. (2024). Effect of *Pleurotus eryngii* on the Characteristics of Pork Patties during Freezing and Thawing Cycles. Foods.

[99] Wang L., Li C., Ren L., Guo H., Li Y. (2019). Production of Pork Sausages Using *Pleurotus eryngii* with Different Treatments as Replacements for Pork Back Fat. J. Food Sci..

[100] Chung S.I., Kim S.Y., Nam Y.J., Kang M.Y. (2010). Development of surimi gel from king oyster mushroom and cuttlefish meat paste. Food Sci. Biotechnol..

[101] Mandliya S., Pratap-Singh A., Vishwakarma S., Dalbhagat C.G., Mishra H.N. (2022). Incorporation of Mycelium (*Pleurotus eryngii*) in Pea Protein Based Low Moisture Meat Analogue: Effect on Its Physicochemical, Rehydration and Structural Properties. Foods.

[102] Nian L., Zhou K., Li Q., Hu X., Su X., Yan X., Tian L., Xu B. (2026). Incorporation of yeast protein improves the textural properties of a fungal meat analogue from *Pleurotus eryngii* mycelium. Innov. Food Sci. Emerg. Technol..

[103] He A., Xu J., Hu Q., Zhao L., Ma G., Zhong L., Liu R. (2023). Effects of gums on 3D printing performance of *Pleurotus eryngii* powder. J. Food Eng..

[104] Lv S., Li H., Liu Z., Cao S., Yao L., Zhu Z., Hu L., Xu D., Mo H. (2024). Preparation of *Pleurotus eryngii* protein baked food by 3D printing. J. Food Eng..

[105] Lv S., Zhu X., Liu Z., Hu L., Xu D., Chitrakar B., Mo H., Li H. (2022). Edible *Pleurotus eryngii* Papery Food Prepared by Papermaking Process. Foods.

[106] Hu Q., Wu Y., Zhong L., Ma N., Zhao L., Ma G., Cheng N., Nakata P.A., Xu J. (2021). In vitro digestion and cellular antioxidant activity of β-carotene-loaded emulsion stabilized by soy protein isolate-*Pleurotus eryngii* polysaccharide conjugates. Food Hydrocoll..

[107] Qian Z., Dong S., Zhong L., Zhan Q., Hu Q., Zhao L. (2023). Effects of carboxymethyl chitosan on the gelling properties, microstructure, and molecular forces of *Pleurotus eryngii* protein gels. Food Hydrocoll..

[108] Chang X., Yang A., Bao X., He Z., Zhou K., Dong Q., Luo W. (2021). An innovative structured fruit (SF) product made from litchi juice, king oyster mushroom (*Pleurotus eryngii*) and gellan gum: Nutritional, textural, sensorial properties. LWT.

[109] Liang J., Ishikawa S.-I. (2026). An emulsion-based tracer solute method for visualizing surface and cross-sectional solute distributions in vacuum impregnated porous food matrices. LWT.

[110] Liu Y., Zhang H., Brennan M., Brennan C., Qin Y., Cheng G., Liu Y. (2022). Physical, chemical, sensorial properties and in vitro digestibility of wheat bread enriched with yunnan commercial and wild edible mushrooms. LWT.

[111] Kim Y.-J., Choi Y.-J., Kim J.-H., Cha J.Y., Kim T.-K., Jung S., Kim D.-H., Choi Y.-S. (2026). Enhancing technofunctional properties of an emulsion-type meat analog formulated with mealworm and TVP: Mushroom incorporation at the optimal concentration. Food Sci. Biotechnol..

[112] Zhao W., Hou J., Dong P., Song C., Zhao S., Cui F., Bi S., Cao J., Cheng Y., Cheng F. (2026). Ergosterol-loaded emulsions stabilized by *Pleurotus eryngii* protein-polysaccharide Maillard conjugates: Preparation, stability, delivery effect, and application in improving the quality of apple slices. J. Food Meas. Charact..

[113] Capuano E. (2017). The behavior of dietary fiber in the gastrointestinal tract determines its physiological effect. Crit. Rev. Food Sci. Nutr..

[114] Reagan-Shaw S., Nihal M., Ahmad N. (2008). Dose translation from animal to human studies revisited. FASEB J..

[115] U.S. Food and Drug Administration (2005). Estimating the Maximum Safe Starting Dose in Initial Clinical Trials for Therapeutics in Adult Healthy Volunteers. https://www.fda.gov/regulatory-information/search-fda-guidance-documents/estimating-maximum-safe-starting-dose-initial-clinical-trials-therapeutics-adult-healthy-volunteers.

[116] Nair A.B., Jacob S. (2016). A simple practice guide for dose conversion between animals and human. J. Basic Clin. Pharm..

[117] Holscher H.D. (2017). Dietary fiber and prebiotics and the gastrointestinal microbiota. Gut Microbes.

[118] Vandeputte D., Falony G., Vieira-Silva S., Wang J., Sailer M., Theis S., Verbeke K., Raes J. (2017). Prebiotic inulin-type fructans induce specific changes in the human gut microbiota. Gut.

[119] EFSA Panel on Dietetic Products, Nutrition and Allergies (NDA) (2011). Scientific Opinion on the substantiation of a health claim related to barley beta-glucans and lowering of blood cholesterol and reduced risk of (coronary) heart disease pursuant to Article 14 of Regulation (EC) No 1924/2006. EFSA J..

[120] 21 CFR § 101.81. Health Claims: Soluble Fiber from Certain Foods and Risk of Coronary Heart Disease (CHD). Code of Federal Regulations, Title 21, Part 101, Section 101.81. https://www.ecfr.gov/current/title-21/part-101/section-101.81.

[121] European Parliament and Council (2015). Regulation (EU) 2015/2283 of 25 November 2015 on Novel Foods, Amending Regulation (EU) No 1169/2011 and Repealing Regulation (EC) No 258/97 and Regulation (EC) No 1852/2001. Off. J. Eur. Union.

[122] European Commission Novel Food Status Catalogue: Entry for Pleurotus eryngii (Dehydrated Mycelium Powder).

[123] European Commission (2025). Commission Implementing Regulation (EU) 2025/691 of 9 April 2025 Authorising the Placing on the Market of Vitamin D_2_ Mushroom Powder as a Novel Food and Amending Implementing Regulation (EU) 2017/2470. OJ L, 2025/691, 10.4. http://data.europa.eu/eli/reg_impl/2025/691/oj.

[124] U.S. Food and Drug Administration GRAS Notice No. GRN 000413: β-Glucans from *Ganoderma lucidum* mycelia. GRAS Notice Inventory, GRN No. 413; U.S. FDA: Silver Spring, MD, USA, 2018; The FDA Responded with No Questions on 10 August 2012. https://www.fda.gov/food/generally-recognized-safe-gras/gras-notice-inventory.

[125] U.S. Food and Drug Administration GRAS Notice No. GRN 001124: β-Glucans from *Hericium erinaceus*. GRAS Notice Inventory, GRN No. 1124; U.S. FDA: Silver Spring, MD, USA, 2018; Evaluation Ceased at the Notifier’s Request on 27 September 2023 (no GRAS Determination Reached). https://www.fda.gov/food/generally-recognized-safe-gras/gras-notice-inventory.

