# Supplementary material for: Pleurotus eryngii as a Source of Candidate Prebiotic Substrates: Formation Routes, Potential Activities and Applications in Food Systems
Source: Foods. 2026 Jul 17;15(14):2527. doi: 10.3390/foods15142527 (PMC13408502; doi:10.3390/foods15142527)
Supplement: Supplementary file 1 [file foods-15-02527-s001.zip › foods-4392135-supplementary.pdf]

Supplementary Materials

Literature search and study selection

Consistent with the narrative-review design of this article, the search below was informed by the reporting-transparency principles of PRISMA but did not constitute a systematic review: no review protocol was registered, and no formal risk-of-bias appraisal or quantitative meta-synthesis was performed.

Databases, coverage and study type. Two databases were searched: the Web of Science Core Collection and PubMed. Only English-language records were considered, and the primary document type was restricted to peer-reviewed original research articles; review articles were additionally retrieved for the background block (Block 6) to provide comparative and definitional context. The coverage window was 1 January 2000–30 April 2026. In Web of Science, searches were run with Timespan 2000–2026, Language = English, Document Types = Article, and Indexes = Web of Science Core Collection. In PubMed, the same window was imposed by appending the following filter to each query: AND ("2000/01/01"[Date - Publication] : "2026/04/30"[Date - Publication]).

Thematic blocks and search strings. Six thematic blocks were searched, and the records retrieved for each block were curated into a separate collection before synthesis. The Web of Science topic-search (TS=) strings used for each block are given below; for PubMed, the equivalent field queries were combined with the publication-date filter above.

Block 1—Basic composition and functional substrates. TS=("Pleurotus eryngii" OR "king oyster mushroom" OR "king trumpet mushroom") AND TS=(polysaccharid* OR beta-glucan OR β-glucan OR glucan OR "dietary fiber" OR "dietary fibre" OR "cell wall" OR oligosaccharid*) AND TS=(composition OR characterization OR structure OR "molecular weight" OR monosaccharid* OR linkage OR physicochemical)

Block 2—Extraction, processing and formation routes. TS=("Pleurotus eryngii" OR "king oyster mushroom") AND TS=(extraction OR purification OR "hot-water extraction" OR enzyme-assisted OR ultrasound-assisted OR microwave-assisted OR fermentation OR "enzymatic hydrolysis" OR processing OR drying OR cooking) AND TS=(polysaccharid* OR beta-glucan OR "dietary fiber" OR "dietary fibre" OR "bioactive compound")

Block 3—Simulated digestion and fecal fermentation. TS=("Pleurotus eryngii" OR "king oyster mushroom") AND TS=("in vitro digestion" OR "simulated digestion" OR "gastrointestinal digestion" OR INFOGEST OR "fecal fermentation" OR "faecal fermentation" OR "colonic fermentation" OR "gut fermentation")

Block 4—Gut microbiota, SCFAs, intestinal barrier and metabolic health. TS=("Pleurotus eryngii" OR "king oyster mushroom") AND TS=("gut microbiota" OR microbiome OR "intestinal microbiota" OR SCFA OR "short-chain fatty acid" OR acetate OR propionate OR butyrate OR Bifidobacterium OR Lactobacillus OR Akkermansia OR Bacteroides OR "intestinal barrier" OR "tight junction" OR mucin OR mucins OR inflammation OR obesity OR glucose OR lipid OR lipids)

Block 5—Food applications and product development. TS=("Pleurotus eryngii" OR "king oyster mushroom") AND TS=("functional food" OR "food application" OR "food matrix" OR formulation OR bakery OR bread OR noodle OR pasta OR biscuit OR beverage OR yogurt OR dairy OR meat OR sausage OR "meat analog" OR "plant-based meat" OR emulsion OR encapsulation OR rheology OR texture OR sensory)

Block 6—Broader mushroom and prebiotic background (reviews). TS=("edible mushroom" OR "mushroom polysaccharide" OR "fungal polysaccharide" OR "Pleurotus") AND TS=(prebiotic OR "gut microbiota" OR microbiome OR "functional food" OR beta-glucan OR "dietary fiber"); for this block the document type was restricted to review articles.

Eligibility criteria. Records were retained when they were (i) peer-reviewed, English-language original research articles (Block 6 additionally admitted reviews) indexed in the Web of Science Core Collection and/or PubMed within the coverage window; (ii) accompanied by an obtainable title, abstract and a DOI or PMID; and (iii) topically relevant to at least one thematic block—addressing *P. eryngii* composition or structure, formation or processing, in vitro digestion or fecal fermentation, gut-microbiota/SCFA/intestinal-barrier/metabolic readouts, or food-application and product development; the background block additionally admitted comparative edible-mushroom, fungal-polysaccharide and prebiotic sources used for mechanistic or definitional context. Records were excluded when they were conference abstracts, conference proceedings, editorials, letters, patents or theses; were not in English; lacked a retrievable title, abstract or DOI/PMID; or were not topically relevant to any block.

Table S1. Complete inventory of formation routes and structural features of *P. eryngii*-derived candidate prebiotic substrates.

Ref.	Substrate / material	Formation / preparation route	Key structural descriptors	Review interpretation
**A. Purified polysaccharides and β-glucans**				
[10]	APEP-A-b purified β-1,6-glucan	0.5 M NaOH extraction; DEAE-cellulose and Sepharose CL-6B purification	Mw: 22.5; 90.1% glucose; branched β-1,6-glucan with O-3 β-1,3/single β-Glcp branches; branching degree 0.18	Defined β-glucan linking structure, digestion, fermentation and in vivo readouts; dose response noted
[11]	Purified 15.9 kDa α-D-galactan-type heteroglycan	Hot-water extraction at 100 °C; Sevag; DEAE Sepharose and Chromdex 200 PG	Crude yield: 1.69; purity 61.91; Mw: 15.9; Gal/Man/Glc/Fuc = 60.66/37.51/1.11/0.72%; 2,6- and 6-linked α-D-Galp with C-2 3-O-methyl-Gal branches	Defined structure linked to microbiota/metabolomics in a mastitis model; SCFA NR
[12]	Purified PEP polysaccharide	Purified polysaccharide used for in vivo fermentation	Mw: 426; 91.25 ± 3.14% purity; Glc 79.11%, Gal 10.37%, Man 5.75%; β-type glycosidic linkages	Defined substrate for in vivo cecal fermentation and immune endpoints; detailed β-glucan content NR
[44]	PEP-0.1-1, PEP-0-1, PEP-0-2 purified fractions	Ultrasound-assisted hot-water extraction; Sevag; DE-52 and Sephadex G-100	Mw: 3.235; 2.041; 23.933; PEP-0.1-1 Gal:Glc 6.63:93.37; PEP-0-2 Fru/Gal/Glc/Xyl/Man = 0.64/16.62/68.56/0.51/13.67; linkages verified in author QC	Recent detailed purified-polysaccharide structural entry; RAW264.7 context and PEP-0-1 monosaccharide naming retained as reported
[37]	Crude PEP and PEP1-A/PEP2-A/PEP3-A	Hot-water extraction at 80 °C; ethanol precipitation; Sevag; DEAE-cellulose-52 and Sephadex G-100	Crude yield: 7.52; purified yields 2.93–3.64; Mw: 497.5–9506; Glc 75.69–82.78%; α/β configurations; 1,3-Glc and 1,6-Gal linkages	Broad structural background from anti-fatigue/antioxidant context
[38]	PEP polysaccharide	Hot-water extraction at 60 °C for 3.5 h; ethanol	Yield: 5.75; 48.77% carbohydrate; 12.32% protein; Glc/Man/Gal =	Foundational digestive-fate and fecal-fermentation substrate; also anchors Table

Ref.	Substrate / material	Formation / preparation route	Key structural descriptors	Review interpretation
		precipitation; freeze-drying	78.32/9.43/8.47%; FT-IR: uronic acid and pyranose rings	2; Mw, linkage and β-glucan content NR
[55]	Cold-water insoluble gel-like β-glucan fraction	Defatting; hot-water extraction at 100 °C for 6 h; ethanol precipitation; freeze-thawing	Yield: 2.5; β-glucan/total glucan: 99% glucose; β-glucan inferred from structure; β-1,3-glucan backbone with single-unit β-1,6 branches at every third residue	Benchmark purified β-glucan structure
[41]	PEAP-1 alkaline polysaccharide	Defatting; water extraction; 0.5 M NaOH extraction; DEAE-cellulose-52 and Sephadex G-150	Crude yield: 6.3; Mw: ~450; PEAP-1 carbohydrate 97%; β-1,3-D-glucan backbone with sparse α-1,6-Gal branches	Alkaline-extracted β-glucan structural benchmark
[26]	Water- and alkali-soluble glucan fractions from stems	Ethanol washing; boiling water extraction; 1 M NaOH extraction; α-amylase starch removal; Sevag/phenol deproteinization	Mw: L1 ~2200; L2 ~2300; β-glucan/total glucan: Non-starch glucans reported; exact β-glucan% by assay NR; L1: branched β-1,3/1,6-glucan–protein complex; L2: linear α-1,3-glucan	Classic extraction–structure–pure-strain screening evidence from mixed *Pleurotus* species study with separable *P. eryngii* data
[27]	P-2a from stalk residue	Hot-water extraction of stalk residue; Sevag; DEAE Sepharose CL-6B and Sepharose CL-6B	Mw: 410; 75.1% sugar, 22.8% protein; Glc 82.40%; 1,3- and 1,6-linked Glc backbone	Residue valorization and stalk-derived polysaccharide entry from antioxidant/cytotoxicity context
[39]	PEP-1 and PEP-2 purified fractions	Hot-water extraction at 80 °C; ethanol precipitation; freeze-thaw deproteinization; DEAE-52 and Sephadex G-100	Crude yield: 5.4; Mw: PEP-1 25.4; PEP-2 463; PEP-2 Glc/Man/Gal = 78.60/10.92/10.48%; 1→3, 1→6 and terminal Glc linkages	Structural background for glucose-rich heteropolysaccharides from cell-assay context

Ref.	Substrate / material	Formation / preparation route	Key structural descriptors	Review interpretation
[40]	WPEP-N-b 3-O-methylated heterogalactan	Hot-water extraction at 100 °C; ethanol precipitation; DEAE-cellulose and Sepharose CL-6B	Mw: 21.4; Gal 43.8%, Man 39.3%, methyl-Gal 11.7%, Glc 9.2%; α-1,6-Gal/3-O-methyl-Gal backbone with β-Man side chains	Illustrates methylated heteroglycan diversity from macrophage cell-model context
[42]	CWEF, HWEF and AEF fractions	Selective extraction: cold water, hot water, autoclave extraction	Mw: CWEF ~20; HWEF ~13; β-glucan/total glucan: Linear β-1,6-glucan and mixed β-glucans reported; exact β-glucan% NR; CWEF: mannogalactan; HWEF: linear β-1,6-glucan; AEF: mixed glucans with triple-helix conformation	Selective extraction/structure example—macrophage cell model; no direct prebiotic readout
[43]	KOMAP alkaline polysaccharide	5% alkali extraction; Sevag; DEAE-cellulose and Sephadex G-200	Crude yield: 9.36; KOMAP 0.85; Mw: 25; Carbohydrate 94.5%; Glc/Man/Ara = 6.2/2.1/2.0; β-1,4-Glcp and β-1,3,6-Manp backbone	Alkaline extract structural diversity from antitumor-model context
[35]	PELPS-A1/A2/A3 glucans	Hot-water extraction; ethanol precipitation; DEAE-32 and Sephadex G-100	Mw: 132–183; β-glucan/total glucan: All-glucose glucans; exact β-glucan% NR; A1 mixed α/β branched glucan; A2 branched β-glucan; A3 linear α-1,6-glucan	Single-variety PELPS glucan-fraction structure entry
[34]	Hot-water and alkali fractions from multiple strains	Microwave-assisted hot-water extraction; alkaline extraction with 1 M NaOH	Yield: Hot-water 2.55–3.84; alkali 3.47–8.48; β-glucan/total glucan: β-glucan structures reported; exact β-glucan% NR; 1,4-α-glucan, 1,6-β-glucan, mannogalactan and 1,3-β-glucan structures	Strain specificity and extraction-route discussion; gut and food-matrix validation remain outside this structural entry
[47]	Heat-treated PEPS fractions	Freeze-drying, oven-drying or boiling; hot-water extraction at 95 °C;	Yield: 7.31, 6.02 and 7.16 g/100 g; Glc-dominant 87.68–89.31%; oven-drying generated insoluble Maillard aggregates	Shows drying/heat processing as formation route—single-strain bifidogenic/soymilk

Ref.	Substrate / material	Formation / preparation route	Key structural descriptors	Review interpretation
		trypsin/H2O2 purification		data; no complex fecal fermentation
[49]	Eight fractions; five structural types	Sequential cold-water, hot-water, enzyme, dilute alkali and concentrated alkali extraction	Total yield: 1.42% on a fresh-weight basis; CWP 0.19%, HWP 0.10%, EAP 0.14%, DAP 0.65%, CAP 0.34%; Mw: 5–2060 kDa; structural-type proportions: β-1,3-glucan 58.8%, β-1,6-glucan 20.6%, α-1,6-galactan 12.7%, α-1,3-mannan 4.9% and α-1,2-mannan 3.0%	Best structural diversity/formation-route anchor
**B. β-Glucan-enriched, aqueous and crude extracts**				
[19]	Water-soluble polysaccharides from two strains	Ultrasonic-assisted cold-water extraction; ethanol precipitation	Mw: 136–679; β-glucan/total glucan: Fruiting bodies: var. *elaeoselini* 22.41 β-glucans; var. *ferulae* 9.51 β-glucans; Branched α-mannans and mannogalactans; strain/variety differences	Strain/variety differences—no direct prebiotic activity
[21]	Aqueous extracts M1/M2A/M2B	Single hot-water extraction or sequential room-temperature + hot-water extraction	Extraction yield: M1 46.03; M2B 12.58; β-glucan/total glucan: M1 β-glucans 37.76 g/100 g; M2B β-glucans 48.19 g/100 g (direct assay); M2B protein 21.23 g/100 g	Practical aqueous-extract material for pure-strain prebiotic screening
[28]	Crude stalk polysaccharides	Extraction from *P. eryngii* stalk ends	Purity 69.92%; Glc/Man/Gal-dominant with Glc 76.28 molar proportion	Stalk-derived gut–liver axis animal substrate—crude extract; no isolated active component
[50]	SPAE aqueous extract powder	Water extraction at 80–90 °C; spray drying at 180 °C inlet/90 °C outlet;	Mw: NR (qualitative: shorter chains than freeze-dried PEP comparator); Uniform spherical particles; FT-IR peak	Processing route showing how spray drying modulates digestibility and in vivo

Ref.	Substrate / material	Formation / preparation route	Key structural descriptors	Review interpretation
		compared with freeze-dried PEP	at 843 cm−1 indicating α-glycosidic bonds	microbiota readouts; polysaccharide-specific attribution limited
[36]	Insoluble dietary fiber	Acid hydrolysis at pH 1.5–2.0 and alkali treatment	Yield: 56.49; β-glucan/total glucan: NA; dietary fiber 85.15%; WHC 5.16 g/g; swelling 4.69 mL/g; cellulose type I crystallinity 42.48%	Dietary fiber-rich fraction connecting substrate recovery with animal-model gut readouts
[48]	Crude PEP and steam-explosion SEPEP	Steam explosion-assisted extraction	Yield: 17.89; Porous sponge-like microstructure; glucose-dominant monosaccharide profile	Processing-induced structural modulation example—no prebiotic/food matrix validation
**C. Whole-powder substrates**				
[33]	Whole powder, digested powder and β-glucan-enriched extract	Whole powder from wheat straw cultivation; simulated digestion; hot-water β-glucan-enriched extract	β-glucan/total glucan: β-glucans: PEWS 38.7; PEWSD 39.8; PEWSE 49.7 (direct assay); α-glucans reduced by digestion; multiple substrate states compared	Whole-matrix and β-glucan-enriched substrates linking substrate state to elderly fecal fermentation
**D. Oligosaccharide-rich hydrolysates**				
[46]	Saccharidic/phenolic/lipophilic hydrolysate extracts	Microwave hydrodiffusion, autohydrolysis and supercritical CO_2 extraction	Autohydrolysis yield: solubilized up to 80% dry weight; β-glucan/total glucan: Extract contained 73% glucan-based oligosaccharides; Autohydrolysis at 210 °C generated mixed oligosaccharide-rich hydrolysate	Innovative extraction route yielding an oligosaccharide-rich hydrolysate
**E. Chemically modified**				

Ref.	Substrate / material	Formation / preparation route	Key structural descriptors	Review interpretation
**polysaccharide derivatives**				
[14]	Purified and selenized polysaccharides	Hot-water extraction; column chromatography; HNO3/sodium selenite modification	Mw: Native 21.617; selenized 19.681; Se 613.87 μg/g; Gal/Glc/Man/Rha = 35.28/30.00/18.43/16.25; triple helix	Chemical selenization/modification example for structure–activity comparison
[52]	Native and sulfated polysaccharides	Hot-water extraction; SO3–pyridine sulfation	Crude yield: 7.68; Carbohydrate 54.28 g/100 g; degree of sulfation 0.69; sulfate groups at C6 of galactose skeleton	Chemical modification background from antioxidant/antibacterial assay context
[51]	Native and sulfated PEPs	Hot-water extraction; chlorosulfonic acid–pyridine sulfation	β-glucan/total glucan: β-1,3-glucans with β-1,6 branches; Sulfation DS 0.12–0.92; water solubility increased from 35% to 56–86%	Degree-of-modification factor for design-factor synthesis
**F. Protein-rich co-extracts**				
[45]	Crude protein extracts co-extracted with carbohydrates	Alkaline, cellulase-assisted alkaline, ultrasound-assisted alkaline and salt extraction	Extraction yield: Salt 44.5; UAA 31.4; AA 27.7; CAA 11.2; FT-IR shows bound carbohydrates/polysaccharides; protein-polysaccharide co-extract	Protein-rich co-extract with carbohydrate-associated features relevant to boundary-material attribution
**G. Submerged-culture exopolysaccharides**				
[30]	Exopolysaccharide fractions Fr-I and Fr-II	Submerged culture optimized with glucose/tryptone at pH 6 and 25 °C	Maximum EPS yield: 5.16 g/L; Mw: Fr-I 40.98; Fr-II 11.14; Fr-I rigid rod; Fr-II lower Mw; Man/Gal-rich	Mycelial/submerged-fermentation substrate-formation example
**H. Boundary case: Se-biofortified**				

Ref.	Substrate / material	Formation / preparation route	Key structural descriptors	Review interpretation
**protein (non-polysaccharide)**				
[15]	Se-enriched protein	Alkaline extraction; acid precipitation; DEAE Sepharose purification	Mw: Protein bands 15, 35, 55, 170; Se-enriched protein 360.64 mg/kg Se; SeMet/SeCys; increased disulfide bonds	Protein/biofortification route adjacent to carbohydrate candidate substrates; included as a structural reference

Notes: Blocks are grouped by substrate class and evidence resolution, not ranked. APEP-A-b, a homogeneous branched β-1,6-glucan; PEP, *Pleurotus eryngii* polysaccharide; SPAE, *Pleurotus eryngii* aqueous extract; PEWS, *Pleurotus eryngii* in whole food matrix form; PEWSD, *Pleurotus eryngii* in in vitro digested form; PEWSE, *Pleurotus eryngii* form rich in β-glucans extract; EPS, exopolysaccharide. NR, not reported. NA, not applicable. Yields are dry-weight percentages unless otherwise specified; submerged-culture EPS yields are reported in g/L; Mw values are reported in kDa unless otherwise specified and are retained as reported.

Table S2. Complete inventory of digestive-fate, fermentation and microbiota-related studies of *P. eryngii*-derived substrates, grouped by evidence model.

Ref.	Substrate / preparation	Model / exposure	Main microbiota, metabolite and host readouts	Relevance to candidate-prebiotic framing
**A. In vitro fecal fermentation studies**				
[20]	Whole powder from wheat straw or wheat straw/grape marc (β-glucan 38.7/42.2%)	24 h static batch fermentation; 2% w/v; donor context: 8 elderly fecal donors	Microbiota/metabolites: Increased *Lactobacillus*; grape marc cultivation increased *F. prausnitzii* and *Roseburia*/*Eubacterium rectale* group; increased total SCFA; butyrate ~45% of total VFAs at 24 h	Microbiota-modulating whole-food substrate—no prior upper-GI digestion; targeted qPCR
[38]	PEP (Glc/Man/Gal 78.32/9.43/8.47%; Mw NR; β-glucan NR)	Simulated saliva/gastric/intestinal digestion; 24 h human fecal fermentation, 1.0 g PEP; donor context: 5 healthy human fecal donors	Microbiota/metabolites: No Mw/free-monosaccharide change during upper digestion; pH 7.64→5.62; total SCFA 52.86 ± 2.01 mM; enriched Firmicutes incl. *Enterococcus*/*Streptococcus*/*Clostridium*; reduced Proteobacteria/Bacteroidetes	Microbiota-accessible substrate (human fecal-fermentation evidence)
[33]	PEWS/PEWSD/PEWSE (β-glucans 38.7/39.8/49.7%)	Simulated digestion; 24 h elderly human fecal fermentation; donor context: 5 healthy older-adult fecal donors	Microbiota/metabolites: All forms increased total SCFA, acetate, propionate and butyrate; whole matrix enriched *Bifidobacterium* spp. and *F. prausnitzii*; *Bacteroides* spp. increased across forms	Candidate prebiotic (elderly fecal-fermentation evidence)
[45]	Crude protein extracts with bound carbohydrates	In vitro digestion + 20 h fecal fermentation; donor context: human fecal donor n NR	Microbiota/metabolites: Resisted gastric digestion; small-intestinal hydrolysis up to 67.90%; AA/UAA increased propionate 24.42/20.57 mM and butyrate 14.78/14.70 mM; AA reduced Bacillota and increased Bacteroidota	Microbiota-accessible substrate—protein–carbohydrate extract; not polysaccharide-only

Ref.	Substrate / preparation	Model / exposure	Main microbiota, metabolite and host readouts	Relevance to candidate-prebiotic framing
[61]	*P. eryngii protein–oat β-glucan complex*	Static digestion + 48 h fecal fermentation; donor context: 7 healthy young adult fecal donors	Microbiota/metabolites: LC-MS: 606 upregulated and 319 downregulated metabolites; enriched tryptophan, histidine and arginine-related metabolites; host/barrier/immune: D-W/F-W fractions restored tight junctions and mucins in Caco-2/HT-29 model; lowered IL-6/TNF-α	Composite-system evidence—external composite; effects not attributable to *P. eryngii* alone
[62]	*P. eryngii protein–oat β-glucan complex; protein purity 60.07%*	INFOGEST digestion; 48 h fecal fermentation; 2:1 protein–oat β-glucan complexes; donor context: 7 healthy volunteers	Microbiota/metabolites: W-PEP-OG showed strongest digestion resistance and best overall fermentation profile; host/barrier/immune: no in vivo host endpoint	Composite-system evidence (protein–oat β-glucan complex; attribution to mushroom β-glucan is limited)
[59]	PEPs, digested products and fermented products	INFOGEST digestion; anaerobic fecal fermentation; HT-29 and DSS mouse model, 0.8 g/kg; donor context: fecal donor n NR	Microbiota/metabolites: Digested/fermented products with mucin reduced LPS-induced IL-1β/TNF-α and upregulated TFF3/MUC2/ZO-1; host/barrier/immune: in mice restored mucins MUC2/MUC5AC and tight junctions ZO-1/occludin/cadherin-1; reduced D-Lac/DAO/LPS	Prebiotic potential—complex mucus interaction mechanism; no human trial
[64]	Freeze-dried whole powder (58.3% carbohydrates; 20.7% protein)	Sequential in vitro digestion + 48 h fecal fermentation; donor context: human fecal donor n NR	Microbiota/metabolites: Lowered pH; generated lower total SCFA than blank; promoted Actinobacteria/*Bifidobacterium* and Bacteroidetes; reduced Proteobacteria/Fusobacteria/Firmicutes	Microbiota-modulating whole-food substrate (whole-powder fecal-fermentation evidence; SCFA profile context-dependent)
[65]	Se-enriched whole powder (90.01 mg/kg Se; mostly SeMet)	Simulated digestion; 48 h fecal fermentation with 1 mg/L Pb; donor context: human fecal donor n NR	Microbiota/metabolites: Se bioaccessibility 84.61%; increased propionate, n-butyrate and total SCFA; increased *Prevotella*/*Bifidobacterium*/*Desulfovibrio*; microbial Pb adsorption 79.06% vs 3.61% Pb control; host/barrier/immune: in vitro lead adsorption; no in vivo host endpoint	Microbiota-modulating whole-food substrate (detoxification-context in vitro evidence)

Ref.	Substrate / preparation	Model / exposure	Main microbiota, metabolite and host readouts	Relevance to candidate-prebiotic framing
[66]	Lyophilized whole powder; NMR: trehalose, choline, nicotinate, organic acids	24 h static elderly fecal fermentation; donor context: elderly fecal donor n NR	Microbiota/metabolites: Increased Lactobacillaceae, Bifidobacteriaceae, Lachnospiraceae, Ruminococcaceae; higher butyrate, propionate, BCAAs, aromatic AAs and GABA	Microbiota-modulating whole-food substrate—no prior upper-GI digestion
[68]	Whole powder (β-glucan 38.7% dry weight)	24 h static fermentation with feces from autistic and neurotypical children; 2% w/v; donor context: autistic and neurotypical child donor n NR	Microbiota/metabolites: Autistic donors: *Bacteroides* spp. 9.23 log10 copies/mL, *F. prausnitzii* 7.32; neurotypical: *A. muciniphila* 4.67; propionate 12.63 µmol/mL and butyrate 19.97 µmol/mL in autistic group	Microbiota-modulating whole-food substrate—single study in special donor group; targeted qPCR
[84]	Fermentation supernatants from whole powder	24 h elderly fecal fermentation; Caco-2 + LPS model; donor context: elderly fecal donor n NR	Microbiota/metabolites: Fermentation supernatants used as post-fermentation bioactive media; host/barrier/immune: upregulated ZO-1, occludin and claudin-1 mRNA after LPS challenge	Prebiotic potential—mRNA-level cell model; no protein validation
[85]	Whole powder, digested powder and hot-water extract (β-glucan 38.7–49.7%)	24 h elderly fecal fermentation; Caco-2 + LPS model; donor context: 5 elderly fecal donors	Microbiota/metabolites: Fermentation supernatant from whole powder restored ZO-1/occludin mRNA and modulated immune genes; host/barrier/immune: mRNA-level barrier/immune response; cytokine protein secretion not detected	Prebiotic potential—independent publication focused on LPS-challenged Caco-2 barrier/immune mRNA readouts; no specific microbiota taxa or SCFA measured in the paper

Ref.	Substrate / preparation	Model / exposure	Main microbiota, metabolite and host readouts	Relevance to candidate-prebiotic framing
[86]	Fecal fermentation supernatant of whole powder	24 h fecal fermentation; 2% v/v supernatant applied to Caco-2 and ex vivo human biopsies; 1 mM sodium deoxycholate stressor; donor context: fecal donor n NR; ex vivo biopsy donors n=10 healthy adults	Microbiota/metabolites: Fermentation supernatant used; microbiota/SCFA not table-level primary readout; host/barrier/immune: Caco-2: ZO-1 0.89, claudin-1 0.78 vs untreated; biopsies: HRP passage 106.07 vs 175.62 fmol/mL with stressor; immune mRNA downregulated in high responders	Prebiotic potential—independent publication focused on SDC-induced Caco-2/ex vivo biopsy barrier and immune readouts; supernatant, not intact substrate
[88]	Fermentation supernatant of whole mushroom powder	24 h elderly fecal fermentation; immune-cell model; donor context: elderly fecal donor n NR	Microbiota/metabolites: Fermentation supernatant increased TNF-α gene expression in immune-cell model relative to controls; host/barrier/immune: cell-model immune readout only	Cautionary immune readout—cell-model-dependent immune readout; pro-inflammatory signal in vitro; not direct in vivo evidence
[89]	Whole powder in fresh durum wheat pasta, 8.62/17.24%	Simulated digestion + 42 h fecal fermentation; donor context: 3 healthy donors	Microbiota/metabolites: pGI lowered to 79.8 at 8.62%; 17.24% reduced protein digestibility to 77.21%; culture-based fermentation supported intermediate bifidobacteria/coliforms; host/barrier/immune: Caco-2 antioxidant protection not strong; no in vivo endpoint	Composite-system evidence (culture-plating readout; metabolite and sequencing readouts not reported)
**B. Animal in vivo studies**				
[10]	APEP-A-b (22.5 kDa branched β-1,6-glucan; 90.1% Glc)	Simulated digestion; 24 h mouse fecal fermentation; mice 100/200/500 mg/kg·d for 14 d; donor context: mouse fecal donor n NR	Microbiota/metabolites: No obvious Mw change in digestion; fermented/degraded by fecal microbiota; 200 mg/kg increased *Lactobacillus*, *Bifidobacterium*, Lachnospiraceae, Rikenellaceae; decreased *C. perfringens*; increased cecal acetate and butyrate; host/barrier/immune: 200 mg/kg increased phagocytosis capacity by 14.8%; 100/500 mg/kg no obvious change	Candidate prebiotic (mouse digestion–fermentation and in vivo evidence; dose response noted)

Ref.	Substrate / preparation	Model / exposure	Main microbiota, metabolite and host readouts	Relevance to candidate-prebiotic framing
[11]	PEP (15.9 kDa α-D-galactan-type heteroglycan; Gal/Man/Glc/Fuc 60.66/37.51/1.11/0.72%)	Mastitis mice; 400/800/1200 mg/kg gavage 7 d; LPS mammary induction; donor context: NA	Microbiota/metabolites: 1200 mg/kg altered Firmicutes/Bacteroidota ratio, decreased *Lactobacillus* and increased *Phocaeicola* A 858004 and *Bacteroides* H; downregulated PGE2, 16(R)-HETE, L-kynurenine, riboflavin-5-phosphate; host/barrier/immune: reduced mammary inflammation; restored blood–milk barrier proteins	Prebiotic potential—Gut–mammary mechanism associative; SCFA and intestinal barrier NR
[12]	PEP (426 kDa; 91.25% purity; Glc 79.11%; β-type linkages)	C57BL/6 mice; up to 0.8 g/kg for 6 weeks; donor context: NA	Microbiota/metabolites: Cecal pH to 7.25; colonic pH to 7.29; increased total SCFA incl. acetate, propionate, butyrate; decreased Firmicutes/Bacteroidetes; increased Lactobacillaceae/Bacteroidaceae; host/barrier/immune: increased spleen/thymus/liver indices; increased intestinal sIgA and serum cytokines	Candidate prebiotic (animal-model immune context; mechanism-resolution priority)
[13]	WPEP (167 kDa β-type glycosidic polysaccharide; Xyl/Man/Glc/Gal 21.35/3.28/73.22/1.63)	DSS colitis mice; 0.2/0.8 g/kg gavage for 44 d; donor context: NA	Microbiota/metabolites: 0.8 g/kg decreased *A. muciniphila* and *C. cocleatum*; increased *B. pseudolongum*, *L. reuteri*, *L. salivarius*, *R. bromii*; host/barrier/immune: DAI 2.78→1.80; colon length 9.31→10.89 cm; reduced mucosal immune cells and cytokines	Candidate prebiotic (mouse colitis model with microbiota, barrier and immune readouts)
[15]	Se-enriched protein (360.64 mg/kg Se; bands 15–170 kDa)	Simulated digestion; Caco-2; fecal fermentation; mice 200 mg/kg/d in lead toxicity; donor context: fecal donor n NR	Microbiota/metabolites: Digest hydrolysis degree 27.65%; fermentation enriched *Megasphaera*/*Mitsuokella*/*Phascolarctobacterium* and decreased *Escherichia*/*Fusobacterium*; microbial Pb adsorption 90.12%; host/barrier/immune: restored colonic tight junction genes; lowered IL-6/TNF-α; reduced tissue lead accumulation	Non-polysaccharide boundary case (Se-enriched protein in digestion, fermentation and lead-toxicity model)

Ref.	Substrate / preparation	Model / exposure	Main microbiota, metabolite and host readouts	Relevance to candidate-prebiotic framing
[71]	Whole *P. eryngii* powder (18.0% fiber; 57.5% carbohydrates; 1.5/3.0% w/w diet)	DSS colitis mice over four DSS cycles; donor context: NA	Microbiota/metabolites: Increased *Adlercreutzia*, *Akkermansia*, *Lactobacillus*, *Anaerostipes*; decreased *Desulfovibrio*naceae; SCFA increased: 1.5% group acetate 1.82-fold and total SCFA 1.43-fold; 3.0% group butyrate 1.56-fold and total SCFA 1.58-fold vs DSS; host/barrier/immune: reduced DAI, colon shortening and colonic cytokines incl. IL-1β, IL-6, IL-17A	Microbiota-modulating whole-food substrate (whole-powder model; isolated β-glucan/polysaccharide attribution limited)
[72]	Whole powder (35.7% dietary fiber; 1/3% w/w HFD diet)	HFD mice for 8 weeks; donor context: NA	Microbiota/metabolites: 3% powder increased cecal acetate 41.77%, butyrate 130.44%, isobutyrate 52.38%, valerate 109.57%, isovalerate 71.57%; enriched *Akkermansia*/*Lactobacillus*/*Bifidobacterium*/*Sutterella*; host/barrier/immune: reduced weight/fat gain, serum lipids, leptin; modulated hepatic lipid genes	Microbiota-modulating whole-food substrate (whole-food model; fraction attribution not isolated)
[28]	Crude stalk polysaccharides (69.92% purity; Glc 76.28 molar proportion)	AFB1-challenged ducks; 1 g/kg dietary inclusion; donor context: NA	Microbiota/metabolites: Increased Bacteroidota, decreased Firmicutes; enriched *Megamonas*/*Bacteroides*/*Phascolarctobacterium*; increased cecal acetate, propionate and butyrate; host/barrier/immune: restored jejunal ZO-1, ZO-2 and Claudin-1; suppressed hepatic TLR4/NF-κB inflammation	Microbiota-accessible substrate—crude stalk extract; poultry toxin model
[50]	SPAE aqueous extract powder (spray-dried; α-glycosidic FT-IR peak 843 cm−1; unpurified)	Dynamic in vitro digestion; ICR mice, 100/400 mg/kg for 5 weeks; donor context: NA	Microbiota/metabolites: Lower reducing sugar release during gastric digestion; cecal pH 6.35 at 400 mg/kg; total SCFA promoted; enriched *Akkermansia*; reduced *Ruminococcus*/*Clostridium*_XVIII; host/barrier/immune: lower serum IL-1β, IL-6, TNF-α; higher IL-10; spleen/thymus indices increased	Microbiota-accessible substrate—unpurified extract; not a pure polysaccharide

Ref.	Substrate / preparation	Model / exposure	Main microbiota, metabolite and host readouts	Relevance to candidate-prebiotic framing
[36]	IDF (56.49% yield; 85.15% dietary fiber; cellulose type I crystallinity)	HFD obesity prevention and relieving mouse models; 0.5/1/2% IDF diet; donor context: NA	Microbiota/metabolites: *Methylobacterium* and *Staphylococcus* increased in all IDF groups vs HFD control; *Lactobacillus* increased only in relieving model; SCFA patterns model/dose-dependent; host/barrier/immune: improved TC/TG/VLDL/HDL and TNF-α/IL-6/IL-1β/IL-10 vs HFD model	Microbiota-accessible substrate—no upper-GI digestion; model-specific comparisons
[54]	Composite fermented sour soybean milk with <3 kDa *P. eryngii* polypeptides	0.2 mL/day gavage for 30 d in CTX immunosuppressed mice; donor context: NA	Microbiota/metabolites: *Bacteroides* 10.88% in high-dose FPEP vs 7.83% model; *Lactobacillus* decreased vs model; SCFA not measured; host/barrier/immune: immune/antioxidant indices improved in composite system	Composite-system evidence—effects belong to fermented soymilk + LAB + polypeptide system
[67]	Hot-water extract (43% total glucans; 32% β-glucans)	24 h fecal fermentation supernatant; mouse gavage 600 mg/kg for 14 d; donor context: NA	Microbiota/metabolites: Fermentation supernatant reduced mitomycin C-induced micronuclei in human lymphocytes by 28.1%; taxa/SCFA not directly characterized; host/barrier/immune: reduced cyclophosphamide-induced bone marrow micronuclei; upregulated gut NrF2, NfκB, DNMT1, IL-22 mRNA	Prebiotic potential—no microbiota taxa or SCFA profiling
[73]	Crude polysaccharide extract; structure/purity/Mw NR	HFD mice; 200/400/800 mg/kg/day for 8 weeks; donor context: NA	Microbiota/metabolites: 400 mg/kg increased cecal acetate 1.40-fold, propionate 1.24-fold, butyrate 22.95%; decreased Firmicutes/Bacteroidota; increased *Ligilactobacillus*, Muribaculaceae_unclassified, *Akkermansia* 3.13-fold; host/barrier/immune: reduced body weight up to 21.14%; restored colonic tight junctions and mucins; lowered colonic cytokines	Microbiota-accessible substrate—administered extract lacks structural characterization

Ref.	Substrate / preparation	Model / exposure	Main microbiota, metabolite and host readouts	Relevance to candidate-prebiotic framing
[77]	PE polysaccharides; structure NR	Acrylamide-treated mice; 100/200/400 mg/kg/day for 6 weeks; donor context: NA	Microbiota/metabolites: 400 mg/kg decreased *Cryptobacteroides*/*Streptococcus* and increased *Ligilactobacillus*, *Lactobacillus*, *Duncaniella*, *Prevotella*, *Acetatifactor*, *Kineothrix*; serum butyric acid +39%; host/barrier/immune: increased IgA/IgM/IgG; decreased Caspase-1, IL-1β, splenic NLRP3/P2X7	Microbiota-accessible substrate—structure not characterized; systemic toxicity model
[78]	PEPF/PEP (Mw ~740 kDa; Glc backbone 82.5%; α/β linkages)	HFD mice; 5% PEPF in diet for 16 weeks; donor context: NA	Microbiota/metabolites: Increased fecal lipid/bile acid excretion; increased *Anaerostipes*, *Clostridium* and *Lactococcus*; decreased *Roseburia* and *Lactobacillus;* GPR43 upregulated; host/barrier/immune: reduced weight gain, mesenteric fat and serum LDL; hepatic LDLR upregulated	Candidate prebiotic—dietary animal model; active fraction attribution limited
[79]	Protein-rich extract (27.7% yield; 48.6% protein purity)	DSS colitis mice; 400/800 mg/kg for 24 d; donor context: NA	Microbiota/metabolites: 800 mg/kg reduced *Escherichia-Shigella* to 5.42% and inhibited *Enterococcus*/*Flavonifractor*/*Lachnoclostridium*; host/barrier/immune: restored ZO-1/occludin; lowered TNF-α/IL-6/IL-1β; raised IL-10 and antioxidant enzymes	Microbiota-accessible substrate (48.6% purity; co-extractives likely contribute)
[76]	Extruded rice composite (20% mushroom powder + 80% rice flour; pGI 60.7)	HFD/STZ diabetic mice; 20% extruded rice in HFD for 8 weeks; donor context: NA	Microbiota/metabolites: Decreased Firmicutes/Bacteroidota; increased *Bacteroides*, *Allobaculum*, *Lactobacillus*, *Akkermansia* and *Parasutterella*; host/barrier/immune: OGTT AUC −37.93%; TC −18.22%, TG −19.65%, LDL-C −52.25%, HDL-C +74.06%; activated hepatic PI3K/AKT	Composite-system evidence—composite food matrix; effects not attributable solely to *P. eryngii*

Ref.	Substrate / preparation	Model / exposure	Main microbiota, metabolite and host readouts	Relevance to candidate-prebiotic framing
**C. Pure-strain and boundary assays**				
[26]	L1/L2 glucans (L1 β-1,3/1,6-glucan-protein complex ~2200 kDa; L2 α-1,3-glucan ~2300 kDa)	Pure-strain cultivation in glucose-free MRS; donor context: NA; 9 pure probiotic strains	Microbiota/metabolites: L1/L2 stimulated *Lactobacillus* growth; L2 doubled growth rate of *Lactobacillus* Lac A; *Bifidobacterium* Bifi B grew only with *P. eryngii* extracts	Prebiotic potential (pure-strain utilization evidence)
[47]	FDPEPS/ODPEPS/BTPEPS (Glc-dominant ~89%; heat-treated)	48 h pure-strain *B. longum* fermentation; donor context: NA; pure *B. longum*	Microbiota/metabolites: Stimulated *B. longum*; viable counts FDPEPS 8.73 log CFU/mL, BTPEPS 8.71, ODPEPS 8.63; pH down to 5.31	Prebiotic potential—single-strain and soymilk-adjacent data; no fecal fermentation
[63]	Irradiation-treated crude polysaccharides	Single-strain *Lactobacillus plantarum*/*Kluyveromyces marxianus* microcalorimetry; donor context: NA; pure strains	Microbiota/metabolites: 1% non-irradiated polysaccharide growth enthalpy 5.687 J/g vs 2% glucose 10.325 J/g; irradiated samples increased growth enthalpy up to 6.681 J/g; *K. marxianus* poorly utilized them	Prebiotic potential (single-strain heat-flow assay)
[87]	PEP and digested products DPEP	3-stage in vitro digestion; mucus interaction for 12 h; donor context: NA	Microbiota/metabolites: DPEP interacted with extracted porcine intestinal mucus, forming an entangled network; zeta −20 to −22.03 mV; altered mucin secondary structure; host/barrier/immune: mucus interaction only; no host physiology	Digestive-fate support (upper-GI digestion and mucus-interaction evidence)

Notes: Blocks are grouped by evidence level, not ranked. APEP-A-b, a homogeneous branched β-1,6-glucan; WPEP, *Pleurotus eryngii* B-type glycosidic polysaccharide; PEP, *Pleurotus eryngii* polysaccharide; SPAE, *Pleurotus eryngii* aqueous extract; PEWS, *Pleurotus eryngii* in whole food matrix form; PEWSD, *Pleurotus eryngii* in *in vitro* digested form; PEWSE, *Pleurotus eryngii* in rich-in-β-glucans extract form; EPS, exopolysaccharide; DPEP, digestion product of *Pleurotus eryngii* polysaccharides. NA, not applicable.

Table S3. Complete product-level inventory of *P. eryngii*-based food applications, grouped by food category and annotated with validation status.

Validation status	Ref.	Food system	*P. eryngii ingredient / level*	Processing or product design	Main technological / nutritional outcomes	Validation context
**A. Bakery, cereal, pasta and staple foods**						
T	[91]	Common wheat pasta	β-glucan-rich fractions, 2/4/6% flour replacement	Pasta dough; sensory, cooking and texture tests	4% best balance; hardness comparable to semolina pasta; 6% disrupted network	Technological/sensory evidence characterization
P-Ferm	[89]	Fresh durum wheat semolina pasta	Whole powder, 8.62% and 17.24%	Pasteurized prototype in modified atmosphere; 4 °C storage	8.62%: fiber 8.7%, cooking loss 5.4%, acceptable sensory; shelf-life stable 110 d	Food composite; gut-related readout is culture-plating-based
P-Cell	[93]	Taralli baked snack	Whole powder, 5% and 10%	Dough rested 4 °C 15 min; baked 200 °C 20 min	10%: 7.91% dietary fiber, 3.03 g/100 g β-glucan; sensory acceptance comparable despite bitterness/astringency	Cell and inflammation-related readouts; microbiota profiling remains a next step
T	[92]	Bread	Freeze-dried whole powder, 5% or 10%	Breadmaking with wheat flour	10% decreased height and increased firmness; sensory overall assessment comparable; added vitamins B12/D3 and biotin	Nutritional/technological characterization
T	[94]	Cookie / wheat dough	Freeze-dried mushroom flour, 5–25%	Dough and baked cookie formulation	25% reduced stability; 15% optimized sensory; >15% lowered flavor/texture	Digestion and microbiota validation not assessed
T	[90]	Sourdough bread	Whole powder, 10% w/w	LAB sourdough, 8 h at 30 °C; baked 200 °C 20 min	Mentana bread: 3.95 g/100 g fiber, 220 mg/100 g β-glucans, 106 mg/100 g	Functional bread composition; gut-health validation not assessed

Validation status	Ref.	Food system	*P. eryngii ingredient / level*	Processing or product design	Main technological / nutritional outcomes	Validation context
					polyphenols, high sensory acceptance	
P-Animal	[76]	Extruded rice	20% whole mushroom powder + 80% rice flour	Twin-screw extrusion at 220 r/min	Medium glycemic index food, pGI 60.7; metabolic/microbiota effects in mice	Composite rice matrix; effects not solely *P. eryngii*
**B. Fermented dairy-like and plant-based systems**						
P-Pro	[47]	Soymilk matrix	PEPS from differently heat-treated mushrooms; 0.5% w/v	Added to soy protein isolate-based soymilk	Promoted *B. longum* growth; FDPEPS/BTPEPS soluble, ODPEPS formed aggregates	Probiotic-growth support in formulation
T	[95]	Fermented milk	Crude PEPS, 0.125–0.5% w/v	12% reconstituted skim milk fermented with *S. thermophilus*	0.5% PEPS maintained starter counts and increased firmness/gumminess but increased whey separation	Starter viability/texture evidence; microbiota validation not assessed
P-Animal	[54]	Fermented sour soybean milk	<3 kDa polypeptides; 0.3/0.5/0.7% groups	Soymilk + lactose/sucrose/gelatin/β-cyclodextrin + mixed LAB; 42 °C, 5.5 h	FPEP LAB count 4.38×10^8 CFU/mL vs 1.84×10^8 SSM; pH 4.26; WHC 53.56%	Composite fermented soymilk system; attribution to the polypeptide component remains limited
P-Pro	[96]	Fermented milk / probiotic survival system	Waste-derived crude polysaccharides; 0.67%	13% skim milk + sucrose; pasteurized; fermented and stored 28 d at 4 °C	Most positively affected starter/probiotic survival during cold storage	Not complex gut fermentation
T	[97]	Yogurt	Tocopherol-rich mycelial extract	Commercial yogurt fortified; 7 d at 4 °C	Nutritional and fatty acid profile stable; antioxidant activity higher than control	Antioxidant-stability evidence characterization

Validation status	Ref.	Food system	*P. eryngii ingredient / level*	Processing or product design	Main technological / nutritional outcomes	Validation context
**C. Meat products and plant-based meat analogs**						
T	[99]	Pork-back-fat replacement sausage	Treated *P. eryngii* cubes replacing the pork-back-fat fraction	Pork back-fat replacement sausage processing	Treatments decreased residual nitrite content; hardness and sensory data reported	Food preservation/quality evidence, not prebiotic evidence
T	[101]	Pea protein low-moisture meat analog	Freeze-dried mycelium, 0–40% w/w	Twin-screw extrusion at 140 °C	30% mycelium improved WHC and expansion; 40% caused structural disintegration	Texture and formulation evidence for meat analog development
T	[100]	Cuttlefish surimi gel	Raw mushroom paste, 0–50% substitution	Steamed at 75 °C for 40 min	Hardness decreased; springiness increased; 40% addition highest sensory score	Surimi texture/sensory characterization
T	[98]	Pork patties	Whole powder, 0–2% meat weight	Repeated freeze–thaw cycles, up to 5 cycles	0.50% reduced thawing loss to 8.57% vs 12.55 control; TBARS 0.66-fold control; higher doses pro-oxidant	Technological stability characterization
T	[102]	Mycelium meat analog	*P. eryngii mycelium + 10% yeast protein*	Freeze–thaw–squeeze; roasted 180 °C for 8 min	10% yeast protein improved hardness, chewiness, fibrous degree and sensory score	Composite meat analog; digestion/microbiota validation not assessed
T	[32]	4D printed mycelium meat analog	Bio-ink with 18% SPI, 5% cassava starch, 6% mycelium	Printed; incubated 10 d at 25 °C/95% RH	Mycelial biomass reached 18.2% dry weight; mechanical hardness increased 6.11-fold	Composite 4D processing characterization
**D. Beverages, instant**						

Validation status	Ref.	Food system	*P. eryngii ingredient / level*	Processing or product design	Main technological / nutritional outcomes	Validation context
**powders and novel structured foods**						
T	[27]	Instant drink	Blanching broth + Fibersol-2; spray-dried powder	Vacuum concentration; spray drying at 150 °C inlet; reconstituted drink	Powders aw 0.283–0.327; best sensory SD13-40 and SD15-40	Composite with commercial dextrin; gut-related data not assessed
T	[103]	3D-printing ink	Freeze-dried powder + food gums; raw powder dietary fiber 20.50%	Gum-water inks; heated 90 °C for 30 min	3% locust bean gum minimized dimensional deviation; improved hardness, viscosity and storage modulus	Composite ink performance characterization
T	[22]	Mushroom chips/snack	Sliced mushroom; UVB-irradiated and baked	Baked 120 °C 20 min; UVB 120 min; seasoning variants	25.43 g/100 g protein; 8.5 g/100 g crude fiber; 32.18% β-glucans; vitamin D2 increased	Snack nutrition and sensory evidence
T	[104]	3D-printed baked potato food	*P. eryngii protein + potato powder + xanthan gum; 20% protein optimum*	Ink cooked 80 °C; printed; baked 150 °C	20% protein optimized extrusion; baking gave sensory score 81.67/100	3D printing functionality characterization
T	[31]	3D-printed mycelium bioconversion matrix	*P. eryngii mycelia on potato starch matrix + 1% xanthan gum*	3D printing and mycelial growth	Starch decreased; β-glucan increased 12.57→24.31%; optimal extrusion with 1% xanthan gum	Bioconversion/processing evidence characterization
T	[105]	Edible papery food	Mycelia, stems, caps or whole fruiting bodies	Papermaking: 4 g dry matter/L water; fluffing/beating; vacuum drying 110 °C, 5 min	Mycelial papery food 36.98% crude fiber and 34.93% protein; stem/mycelia highest palatability	Novel food matrix; digestion/fermentation validation not assessed

Validation status	Ref.	Food system	*P. eryngii ingredient / level*	Processing or product design	Main technological / nutritional outcomes	Validation context
**E. Emulsions, encapsulation and delivery systems**						
P-Dig	[106]	β-carotene emulsion / delivery system	SPI–PEP conjugate; β-carotene-loaded emulsion	High-pressure microfluidization at 90 MPa	Encapsulation efficiency 65.13%; stable pH 4.0–11.0; intestinal FFA release 89.73%	Delivery-system digestion evidence; colonic fermentability of the *P. eryngii* component remains to be tested
T	[53]	Fermented extract ingredient	*L. plantarum-fermented P. eryngii extract*	Bacterial–fungal fermentation	Enhanced antibacterial, antioxidant and anti-allergic bioactivities in vitro	Functional extract, not final food product
T	[107]	Protein gel	*P. eryngii protein + 0–5% carboxymethyl chitosan*	16% protein dispersion heated 90 °C for 30 min	3.0% CMCS maximized hardness, springiness, WHC and storage modulus	Composite gel technology; no biological validation
T	[108]	Structured fruit	Freeze-dried mushroom stem + litchi juice + 0–0.5% gellan gum	Immersion and heating at 90 °C for 30 min	0.25–0.5% gellan restored hardness close to fresh litchi; ~70% sensory similarity	Texture/nutrition product; gut validation not assessed
T	[109]	Vacuum-impregnated porous food carrier	Fresh stipe tissue + 50% v/v oil-in-water emulsion	Vacuum impregnation at −60 to −90 kPa	Pressure influenced core penetration and solute distribution	Physical tracer model; physical-tracer evidence

Notes: Validation status refers to conclusion generated with the final formulated food. Codes: T, technological/nutritional characterization; P-Ferm, partial culture-based fermentation; P-Dig, partial simulated digestion; P-Cell, partial cell, barrier or inflammation-related readouts; P-Animal, partial animal-model microbiota readouts after product consumption; P-Pro, partial probiotic growth/survival or simulated-GI tolerance. PEPS, *Pleurotus eryngii* polysaccharide; FDPEPS, freeze-dried form of *Pleurotus eryngi* polysaccharides; BTPEPS, boiling-treated form of *Pleurotus eryngi* polysaccharides; ODPEPS, oven-dried form of *Pleurotus eryngi* polysaccharides; FPEP, fermented with PEP (*Pleurotus eryngii* polypeptides with a molecular weight of < 3 kDa).
